# Analysis of the DNA-Binding Activities of the Arabidopsis R2R3-MYB Transcription Factor Family by One-Hybrid Experiments in Yeast

**DOI:** 10.1371/journal.pone.0141044

**Published:** 2015-10-20

**Authors:** Zsolt Kelemen, Alvaro Sebastian, Wenjia Xu, Damaris Grain, Fabien Salsac, Alexandra Avon, Nathalie Berger, Joseph Tran, Bertrand Dubreucq, Claire Lurin, Loïc Lepiniec, Bruno Contreras-Moreira, Christian Dubos

**Affiliations:** 1 Institut Jean-Pierre Bourgin (IJPB), INRA, AgroParisTech, CNRS, Saclay Plant Sciences, Université Paris-Saclay, Versailles, France; 2 Estación Experimental de Aula Dei/CSIC, Zaragoza, Spain; 3 Fundación ARAID, Zaragoza, Spain; 4 Paris-Saclay Institute of Plant Sciences (IPS2), UMR INRA-CNRS-P11-P7-UEVE 1403, Saclay Plant Sciences, Evry, France; 5 Biochimie et Physiologie Moleculaire des Plantes, UMR 5004, INRA/CNRS/SupAgro-M/UM, Montpellier, France; Oak Ridge National Laboratory, UNITED STATES

## Abstract

The control of growth and development of all living organisms is a complex and dynamic process that requires the harmonious expression of numerous genes. Gene expression is mainly controlled by the activity of sequence-specific DNA binding proteins called transcription factors (TFs). Amongst the various classes of eukaryotic TFs, the MYB superfamily is one of the largest and most diverse, and it has considerably expanded in the plant kingdom. R2R3-MYBs have been extensively studied over the last 15 years. However, DNA-binding specificity has been characterized for only a small subset of these proteins. Therefore, one of the remaining challenges is the exhaustive characterization of the DNA-binding specificity of all R2R3-MYB proteins. In this study, we have developed a library of *Arabidopsis thaliana* R2R3-MYB open reading frames, whose DNA-binding activities were assayed *in vivo* (yeast one-hybrid experiments) with a pool of selected *cis*-regulatory elements. Altogether 1904 interactions were assayed leading to the discovery of specific patterns of interactions between the various R2R3-MYB subgroups and their DNA target sequences and to the identification of key features that govern these interactions. The present work provides a comprehensive *in vivo* analysis of R2R3-MYB binding activities that should help in predicting new DNA motifs and identifying new putative target genes for each member of this very large family of TFs. In a broader perspective, the generated data will help to better understand how TF interact with their target DNA sequences.

## Introduction

The control of growth and development of all living organisms is a complex and dynamic process that requires the harmonious expression of numerous genes (several thousands in eukaryotes). Regulation of gene expression is thus central to all organisms and it is mainly orchestrated by the activity of sequence-specific DNA binding proteins called transcription factors (TFs). The role of TFs is to modulate gene expression in response to external (*e*.*g*. abiotic and biotic stresses) and internal (*e*.*g*. nutrition or development) signals. TFs can be involved in transcriptional activation, repression or both. TFs possess a modular structure generally comprising a DNA-binding domain (DBD) together with a regulatory or sensing domain. Specific signatures present at the amino acid level (notably into the DBD) have allowed categorizing TFs into various families [1].

Amongst the various classes of TFs the MYB superfamily is one of the largest and most diverse [2]. MYB TFs are widely distributed in all eukaryotic organisms and have considerably expended in the plant kingdom [3]. The MYB domain that characterizes this class of TFs is composed by approximately 50 amino acids, with up to 4 repeats (R) in tandem [4]. This domain is responsible for the binding of MYB proteins to their target DNA sequences. The interaction involves a helix-turn-helix structure that contains three evenly spaced tryptophan residues. These residues form a hydrophobic core which defines the protein fold that ultimately drives recognition of specific DNA sequences. The MYB proteins are gathered into different groups according to the number of repeat(s) found in the MYB domain [3, 5, 6].

In plants, most MYB TFs belong to the R2R3-MYB family (two repeats). For example, out of the 196 *MYB* genes that are present in the *Arabidopsis thaliana* genome, 126 encode R2R3-MYB proteins [3, 7]. R2R3-MYBs are specific to the plant kingdom and are involved in the transcriptional control of plant-specific processes [7]. In parallel to the identification of their different roles *in planta*, researchers have been producing more and more molecular data regarding the R2R3-MYB family over the last 15 years. These data include insights into their expression profiles, the mechanisms that control their activities (*e*.*g*. post-translational modifications, interacting protein partners) or the identification of some of their direct targets [8–11].

R2R3-MYBs control the expression of their target genes through the interaction with specific DNA sequences usually present upstream of the transcribed region within the promoters [12]. These *cis*-regulatory elements have been initially categorized into two main groups, the MYB-core *[C/T]NGTT[G/A]* (subdivided into two types: type I and type II that correspond to the *CNGTT[G/A]* and *TNGTT[G/A]* canonical sequences, respectively) and the *AC*-elements that are *AC*-rich sequences (consensus sequences: *ACC[A/T]A[A/C][T/C]* and *ACC[A/T][A/C/T][A/C/T]*; [13, 14]). Unrelated *cis*-DNA sequences from which specific R2R3-MYBs regulate the expression of their target genes have also been identified in Arabidopsis and other plant species. This is for example the case of Arabidopsis AtMYB88 and AtMYB124/FLP (FOUR LIPS), which recognise the *[A/T/G][A/T/G]C[C/G][C/G]* consensus sequence [15], or the Apple (*Malus domestica*) MdMYB10 that interacts with the *ACTGGTAGCTATT* DNA motif [15, 16].

The identification of these canonical DNA sequences has mainly been achieved through two main approaches. The first one is based on the structural analysis of the promoter of genes whose expression is directly regulated by a specific R2R3-MYB (*e*.*g*. [17]). The second approach is based on *in vitro* analysis of the interaction between a given R2R3-MYB protein (or its DBD) and a pool of random DNA fragments (CASTing and SELEX approaches; [18, 19]). New powerful methods have been developed to identify the DNA motifs that are targeted *in vivo* by virtually any TFs. The main approach relies on the development of ChIP (Chromatin immunoprecipitation) methodology allowing genome-wide identification of the binding sites recognised by a specific TF [20]. Another approach relies on the use of protein-binding microarrays on which interactions between a TF protein and a set of predetermined DNA oligonucleotides are assayed [21]. The use of bioinformatic resources to search for *cis*-regulatory sequences conserved in co-regulated genes coupled with large-scale yeast one-hybrid (Y1H) experiments using an ordered TF library has also been used as an alternative method [22].

Altogether these approaches have allowed the identification of at least one *cis*-element for about one third (44 genes) of the R2R3-MYBs encoded in the genome of *A*. *thaliana* (S1 and S2 Tables). Therefore, one of the remaining challenges in characterizing this large family of TFs is to determine the binding specificity of all R2R3-MYB proteins (or their DBDs). In this regard pioneer work has been made about 15 years ago [14]. It was then proposed that MYB-core type I (*CNGTT[G/A]*) sequences are specific to R2R3-MYB proteins belonging to clade A (*i*.*e*. subgroups 21 and 22, and probably 23) whereas clade B R2R3-MYBs (*i*.*e*. subgroup 18) interacted equally with either type I and type II (*TNGTT[G/A]*) MYB-core sequences. All the other R2R3-MYBs (clade C) were considered as more specific to *AC*-rich sequences (*ACC[A/T]A[A/C][T/C]*). However, a substantial amount of data gathered from various studies revealed that this classification was not fully accurate (S1 and S2 Tables).

In this study we have developed an almost complete collection of *A*. *thaliana* R2R3-MYB open reading frames (ORFs) using the Gateway® technology with and without the stop codon in order to keep the field of application of this library as broad as possible (*e*.*g*. possible ORF mobilisation in various vectors using both, N- and C-terminus gene fusions). Binding activities were assayed *in vivo* in Y1H experiments with a pool of 16 DNA sequences. These DNA motifs are well-characterised *cis*-regulatory elements, known to interact with R2R3-MYB proteins, that belong to both, the MYB-core and *AC*-rich groups [14, 17, 23–26]. Altogether 1904 potential interactions were assayed, leading to the discovery of specific patterns of interactions between the various subgroups of R2R3-MYBs and their DNA target sequences.

## Results

### Large-scale yeast one-hybrid assay

119 individual *Arabidopsis thaliana* R2R3-MYB transcription factor (TF) open reading frames (ORFs) were successfully cloned (with and without the stop codon), which corresponds to a success rate of 94.4%. 16 DNA motifs were assayed (see S3 Table for details) against these 119 R2R3-MYBs, leading to the identification of 1124 positive interactions out of 1904 tested (summarized S4 Table). We estimated the reliability of the Y1H results by comparing them to published results. In total, out of 78 published positive interactions, 57 were confirmed in our experiment. Similarly, from 15 reported negative interactions, we confirmed 9 of them. Overall, these numbers suggest that the precision (proportion of true positive) of the Y1H experiment is 0.90, and its recall 0.73 (true positive rate). We have also estimated the false positive rate, which is 0.092. These parameters indicated that the dataset we have generated was sufficiently robust to be further analysed. The differences observed between the present Y1H screen and the data gathered from the literature most probably reflect the fact that the interactions between the R2R3-MYBs and their DNA targets had been assessed using various *in vitro* and *in vivo* methods [13, 14, 17, 21, 22, 27]. This later point being highlighted by the fact that out of the 21 positive interactions that were not confirmed in this study, 14 were issued from EMSA or oligo arrays.

**Fig 1:**
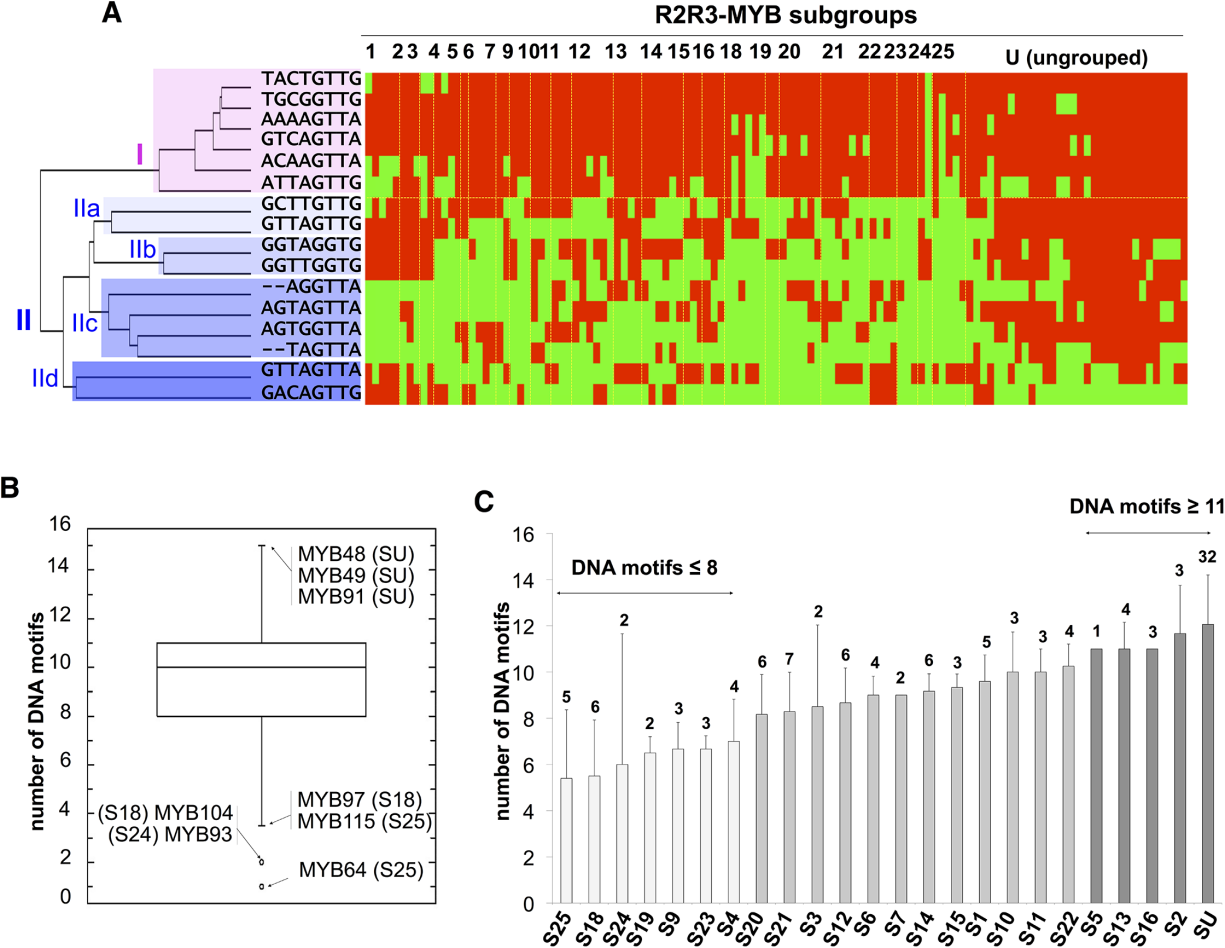
R2R3-MYBs binding activity **(A)** Heat map representation (using the EPCLUST Tool) of the Y1H results. DNA motifs are grouped accordingly to their selectivity against the different R2R3-MYB subgroups. Yellow: yeast growth on selective media (*i*.*e*. interaction between a given DNA motif and a R2R3-MYB), blue: no yeast growth on selective media (*i*.*e*. no *trans*-activation). In pink and blue are highlighted group I and group II DNA motifs, respectively. **(B)** Box plot representation of the number of DNA motifs recognised per R2R3-MYB. **(C)** Number of DNA motifs recognised per R2R3-MYB subgroup. Error bars: binding variation amongst the R2R3-MYBs within each subgroup. Numbers above each column indicate the number of R2R3-MYB of each subgroup. S: subgroup. U: ungrouped.

Hierarchical clustering analysis of the 1904 tested interactions (using the EPCLUST tool; [28]) revealed that the *cis*-elements could be divided into two groups (Fig 1A). The first group (group I) was gathering DNA sequences that were interacting with most R2R3-MYBs (87% of them on average). The others DNA sequences fell in a more specific group (group II, average interaction: 40%) composed of four subgroups whose members displayed in average a similar number of Y1H interactions (IIa: 56%, IIb: 45%, IIc: 34% and IId: 32%).

### Investigating R2R3-MYB binding specificities in yeast one-hybrid assays

We found that most of these R2R3-MYBs have the ability to recognise a variety of DNA motifs; each TF interacting in average with 9.5 (59%) of the selected DNA motifs (S4 Table). We also observed that the variation in the interactions amongst the individual R2R3-MYB and the DNA motifs was considerable (Fig 1B), ranging from one (AtMYB64) to 15 (AtMYB48, AtMYB49 and AtMYB91). Similarly, a wide variation in the interaction capacity was also observed at the R2R3-MYB subgroup level (Fig 1C).

The search of positive (or negative) associations between a specific DNA motif from group II (*i*.*e*. the most discriminating) and at least 75% of the R2R3-MYBs of a given subgroup revealed interesting and unsuspected patterns of interactions (S1 Fig). One striking example was found when focusing on the interaction patterns of R2R3-MYB subgroups 1, 2, 3 and 13. A positive association was found for S2, S3 and S13 with DNA group IIa and IIb, and for S1 with DNA group IIb and IId (S1A, S1B and S1D Fig). These observations most probably indicate that S1, S2, S3 and S13 R2R3-MYBs have some specificity towards the *AC*-rich *cis*-regulatory sequences. This is supported by the fact that two consecutive DNA motifs from group IIa contain an *AC*-rich sequence (*[G/A]CCAAC*) at their junction, which is similar to well described *AC*-II element (*ACCAAC*; [29]). Similarly, two consecutive group IId DNA motifs display an *AC*-rich-like sequence. Still in support of this hypothesis, the detailed study of AtMYB61 binding capacity, which belongs to subgroup 13, clearly demonstrated its strong affinity for the *AC*-rich regulatory sequences [19, 30]. In contrast, no clear positive association with particular R2R3-MYB subgroups was found for motif group IIc (S1C Fig).

### R2R3-MYB binding specificities in relation with their biological roles

**Fig 2:**
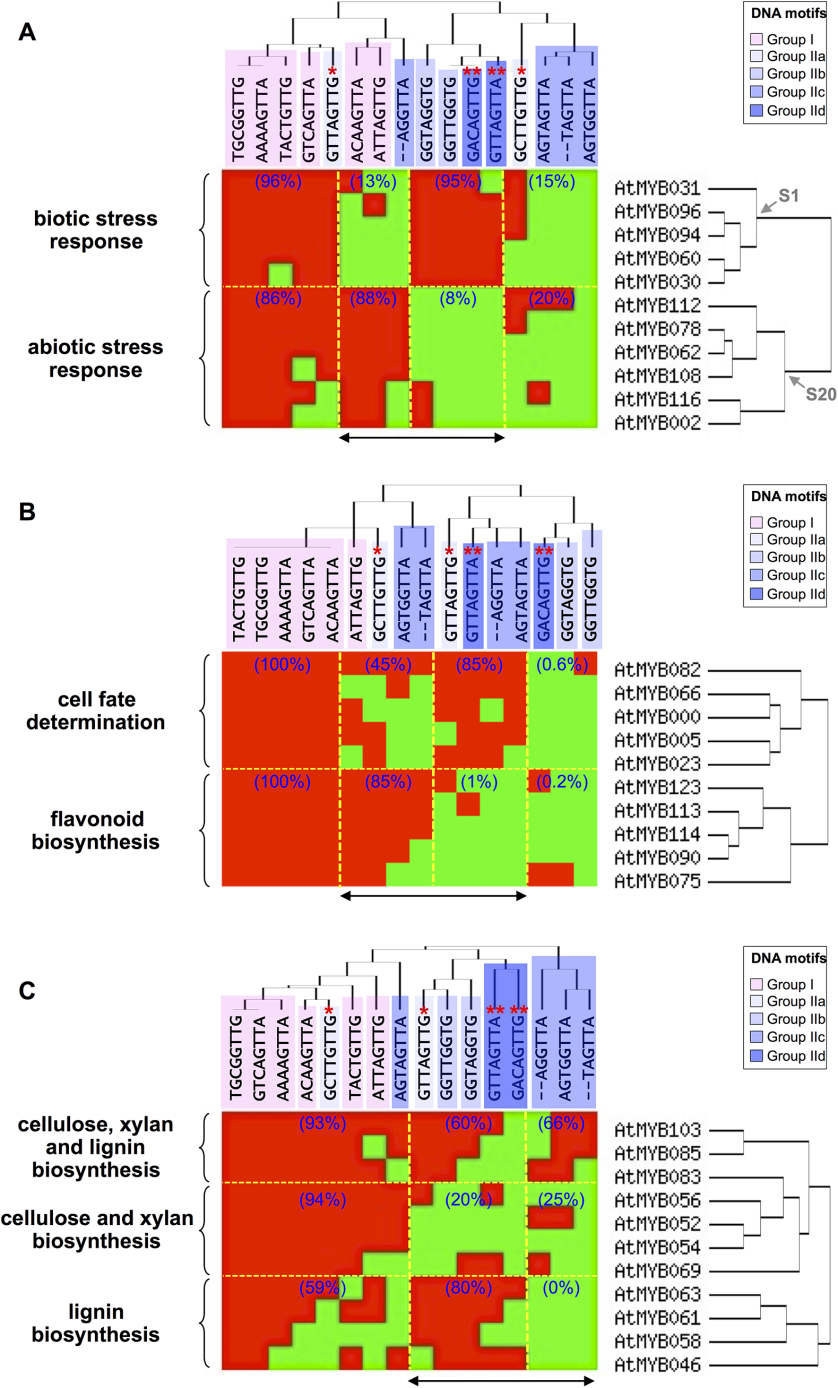
Binding specificities of selected R2R3-MYBs in relation with their biological roles. Heat map representation of the Y1H results observed with selected R2R3-MYBs involved in (**A**) biotic and abiotic stress responses, (**B**) cell fate determination and flavonoid biosynthesis (in TTG1-dependent complexes) and (**C**) cell wall biosynthesis (cellulose and xylan *vs* lignins). Yellow: yeast growth on selective media, blue: no yeast growth on selective media. Stars indicate the DNA sequences that form an *AC*-rich element in between two consecutive DNA motifs (*: group IIa, **: group IId). Double head arrows indicate the most discriminating DNA motifs between the R2R3-MYB groups.

As we found that some R2R3-MYB subgroups displayed specific patterns of interaction with particular DNA motifs, and because the role played *in planta* by numerous R2R3-MYB has been identified [7], we decided to analyse the data from this later point of view (Fig 2). To this end subsets of R2R3-MYB for which biological functions have been well characterized were chosen.

We first investigated R2R3-MYBs belonging to two subgroups associated with plant responses to environmental stresses [31–35], namely S1 and S20 (Fig 2A). We found that these two R2R3-MYB subgroups were displaying striking binding differences toward seven DNA motifs. Those TF from S1 were strongly interacting with the four DNA motifs from groups IIb and IId (95% of positive interactions). It is noteworthy that these four DNA motifs, as well as group IIa elements, contain an *AC*-rich sequence either in the element itself or in between two consecutive sequences. This observation supports the hypothesis that R2R3-MYBs belonging to S1 interact strongly with *AC*-rich sequences. In contrast, S1 R2R3-MYBs were preferentially not interacting with the three other DNA motifs (13% of positive interactions). Interestingly, R2R3-MYBs belonging to S20 were displaying an opposite binding pattern.

We then focused the analysis on the R2R3-MYB subgroups whose activity depends on R/B-like bHLH TFs (subgroup IIIf; [36]) and TTG1 (Fig 2; [37]). These R2R3-MYBs have been described as mainly involved in the transcriptional control of cell fate determination or flavonoid metabolism and belong to subgroup 15 (in addition with the closely related AtMYB82; [38]) and subgroups 5 and 6, respectively [39]. AtMYB5, which in this regard plays a dual role, was also included in the analysis [40]. Interestingly, we found two sets of DNA motifs (composed of a mix of sequences from different groups) that were discriminating between the R2R3-MYBs. The first one was composed of four DNA motifs (from groups IIa, IIc and IId) that were mostly interacting with the R2R3-MYBs involved in the transcriptional regulation of secondary metabolism. The second group (composed of DNA sequences from groups I, IIa and IIc) was even more discriminating as it was almost exclusively associated with TFs involved in the control of cell fate determination. It is noteworthy that AtMYB5 falls into the cell fate determination group when considering its sole DNA binding properties. This later observation is in agreement with previous work that had shown that AtMYB5 transcriptional activity is weaker than that of TT2 when considering the control of the expression of genes involved in proanthocyanidin biosynthesis [10, 41].

Finally, we investigated whether specific patterns of interaction existed between the analysed DNA motifs and R2R3-MYBs involved in secondary cell wall biosynthesis (Fig 2C). Secondary cell wall is a complex structure that contains various compounds including cellulose and xylan (polysaccharides) or lignin (phenolics) whose biosynthesis and accumulation is controlled at the transcriptional level by various R2R3-MYBs that form an intricate regulatory network [42]. We first found that these R2R3-MYBs could be separated into three groups. The first group included AtMYB83, AtMYB85 and AtMYB103, three proteins that mostly play a dual role in secondary wall biogenesis as they regulate cellulose and xylan as well as lignin biosynthesis [43–45]. The second group was composed of genes mainly involved in the transcriptional control of cellulose and/or xylan biosynthesis. This group corresponds to four phylogenetically close members of S21, namely AtMYB52, AtMYB54, AtMYB56 and AtMYB69. Finally, the third group corresponded to genes preferentially involved on transcriptional regulation of lignin biosynthesis (AtMYB46, AtMYB58, AtMYB61 and AtMYB68). We observed that the TFs mostly involved in the regulation of cellulose and xylan biosynthesis were mainly interacting with DNA motifs from group I (93% and 94% of positive interactions *vs* 59%). This feature was even more striking for S21 as they were nearly exclusively interacting with these DNA sequences (82% of all the positive interaction observed for this R2R3-MYB subgroup). In contrast, AtMYB83, AtMYB85 and AtMYB103 were less discriminating as they were displaying a high level of interaction with all type of DNA motifs tested. Finally, the R2R3-MYBs mostly involved in the transcriptional regulation of lignin biosynthesis were more specifically interacting with the DNA motifs containing an *AC*-element (group IIb) or closely related DNA sequences (80% of positive interactions).

### Study of target recognition by R2R3-MYB DNA-binding domains (DBD)

**Fig 3:**
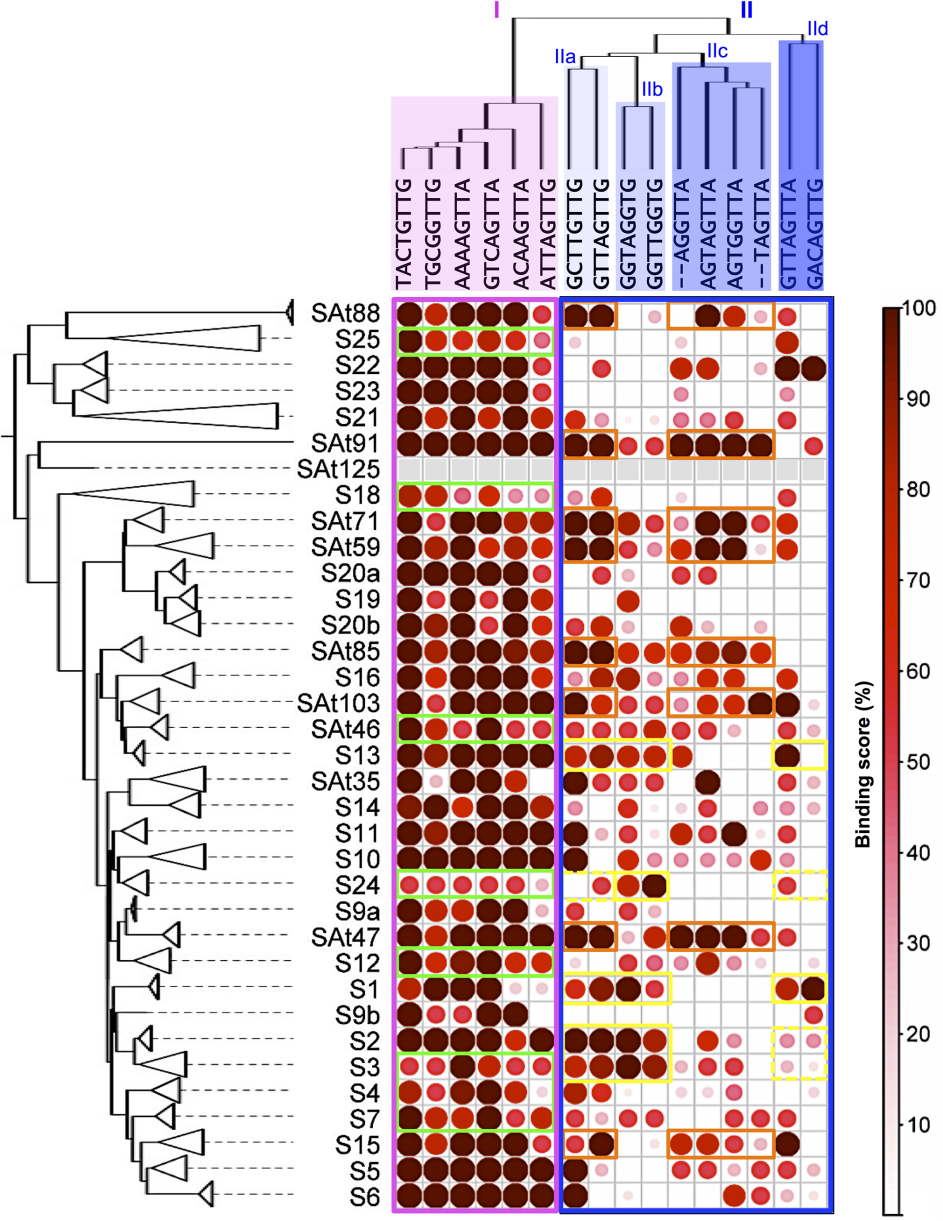
Summary of Y1H results for all R2R3-MYB DNA binding domain (DBD) tested. On the left a maximum likelihood tree that defines a total of 35 DBD subgroups is displayed with collapsed branches. This tree was computed based on a multiple alignment of the R2R3 domains as defined [3]. On top a cladogram of the 16 *cis*-elements assayed is plotted. In pink and blue are highlighted group I and group II DNA motifs, respectively. Binding scores in the matrix take values from 0 to 100% when all members of a subgroup bind to a given DNA sequence with high affinity (++ in S4 Table). Yellow square: low binding scores within DNA group I. Orange and red squares highlight the preferential binding of some DBDs toward the DNA motifs from groups IIa and IIc or groups IIa and IId, respectively.

Because TFs interact with their target DNA sequences through their DNA-binding domain (DBD), we decided to characterize the Y1H interaction patterns through the sole DBD. For this purpose a new phylogenetic analysis has been carried out using the R2R3 MYB domains (Fig 3 and S2 Fig). As a result 35 R2R3-MYB DBD subgroups were defined. Most of the previous R2R3-MYB subgroups were conserved, even if some subgroups were either implemented with additional R2R3-MYB DBDs (S3, S4, S5, S11, S19, S21 and S25) or split in two (S9 and S20). 10 new subgroups (namely SAt35, SAt46, SAt47, SAt59, SAt71, SAt85, SAt88, SAt91, SAt103, and SAt125) were also defined. Interestingly, these subgroups have been independently identified in a recent study where R2R3-MYB proteins from five plant species were analysed together [46].

A binding score [47] was then attributed to each DBD subgroup which reflects the number and the strength of the interactions observed in Y1H experiment with a specific DNA motif; a 100% BS indicates that all the DBDs of a given subgroup interact strongly with a given DNA motif (Fig 3). As expected, a high BS was globally observed for all the DBD subgroups with DNA motifs from group I. S18 and S25 (that display an overall low number of interaction) together with S3, S4, S7, S12, S24 and SAt46 (that weakly interact with this DNA motif group) were the exceptions. While focusing on DNA motifs from group II we identified distinct patterns of interaction suggesting that some cross affinity between specific DBD subgroups and different DNA motifs from group II may exist.

**Fig 4:**
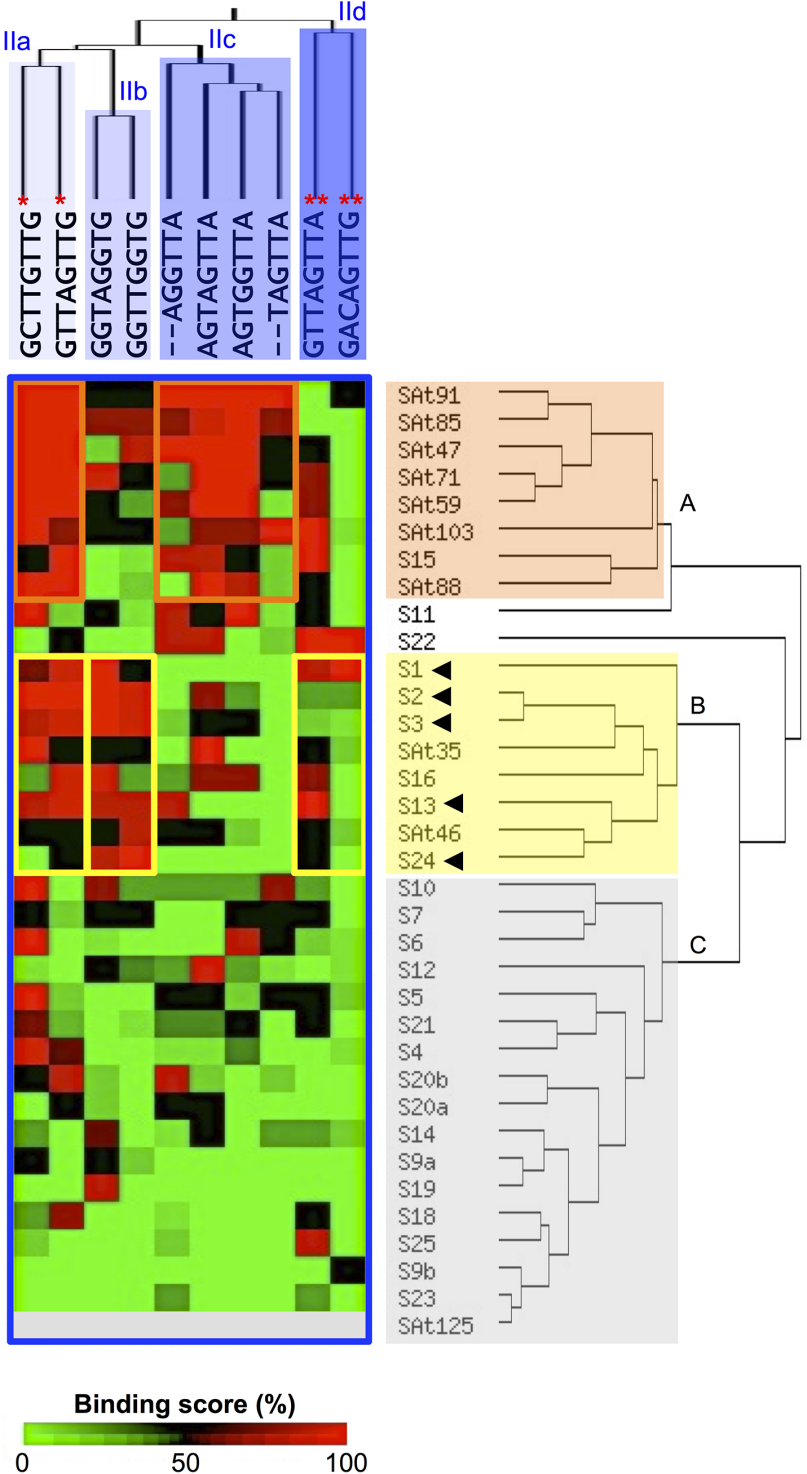
Hierarchical clustering analysis of the binding scores associated with the 35 DNA-binding domains toward the DNA motifs belonging to group II. Preferential binding of DBDs toward groups IIa and IIc or groups IIa and IId are highlighted by orange and red squares, respectively. Stars indicate the DNA sequences that form an *AC*-rich element in between two consecutive DNA motifs (*:group IIa, **: group IId). Arrowheads indicate R2R3-MYB DBDs that were found to be the most strongly associated with group IIb (*AC*-elements) DNA motifs in Fig 3.

This observation was confirmed by hierarchical clustering analysis, highlighting two specific patterns of interaction (Fig 4). The first one comprises DBDs subgroups that were mostly associated with DNA motif from group IIa and IIc (namely S15, SAt47, SAt59, SAt71, SAt85, SAt88, SAt91 and SAt103), suggesting a preferential affinity to MYB-core type II DNA sequences. The second includes DBDs subgroups (namely S1, S2, S3, S13, S16, S24, SAt35 and SAt46) mostly associated with DNA motifs from group IIa and IIb. The data suggest that these later DBD subgroups preferentially bind *AC*-rich sequences with different degrees of specificity, from which S1, S2, S3, S13 and S24 are the more specific.

### Distinguishing features of R2R3-MYB DBDs

**Fig 5:**
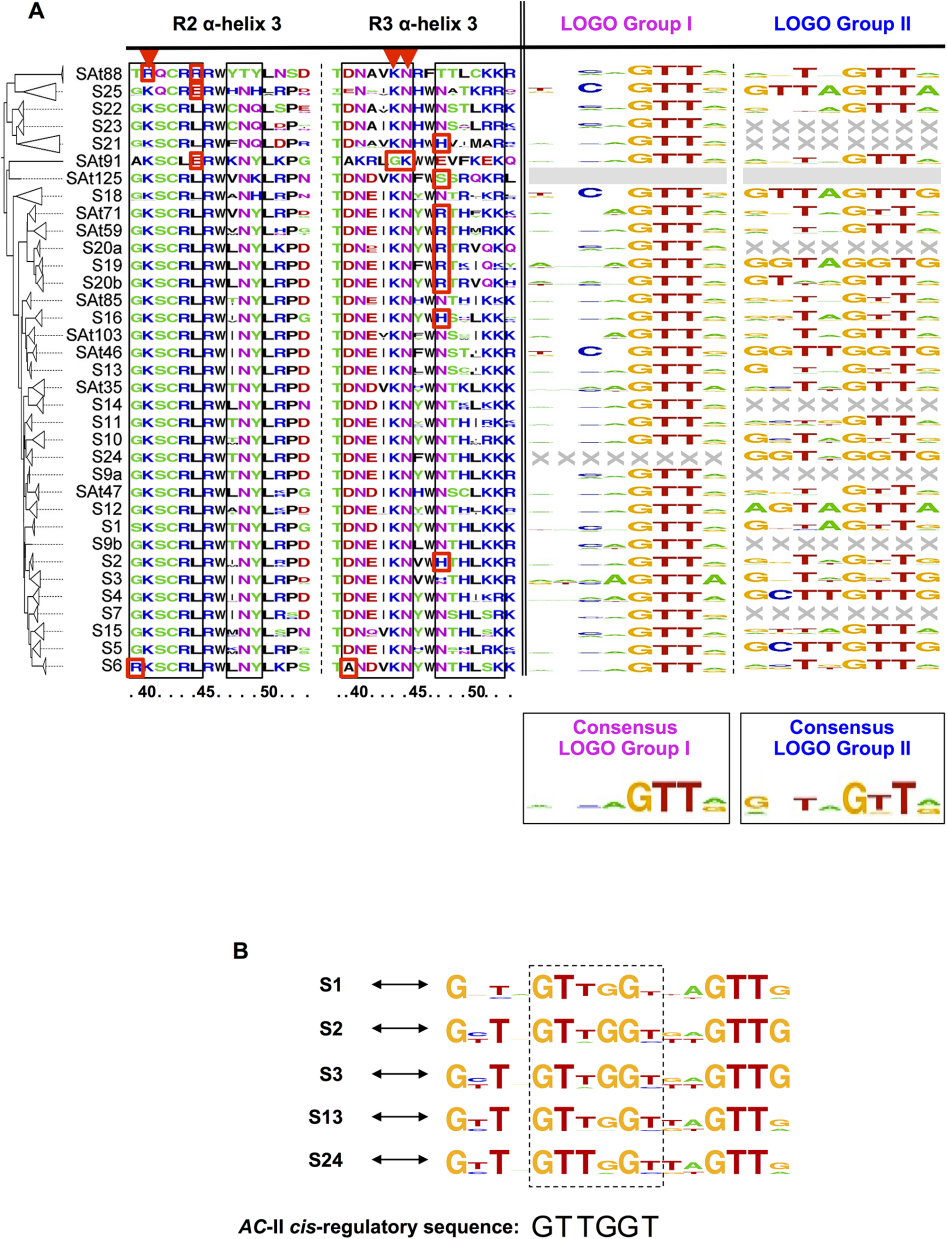
Sequence LOGOs analysis. (**A**) Sequence LOGOs of the R2 and R3 DNA-recognition α-helices of *Arabidopsis thaliana* R2R3-MYB DBD subgroups (left) and of the bound *cis*-elements assayed on the Y1H experiments, classified into two groups (right). The bottom LOGOs are the calculated *consensi* for both groups. Boxed columns within the recognition helices highlight residues that most likely contact DNA nitrogen bases based on alignments to MYB protein data bank structures. Red arrowheads indicate key amino acid residues involved in the interaction with DNA that are conserved in almost all plant and animal MYB proteins ([50, 54]). Red squares highlight key amino acid residues that are associated with specific DBD subgroups and for which some experimental evidences (*in vitro* and/or *in vivo*) on their role in the interaction with DNA targets are available. **(B)** *AC*-rich sequence LOGOs associated with the *trans*-activation activity of DBD subgroup 1, 2, 3, 13 and 24.

MYB proteins dock to the major grove of DNA through the third α-helix of both R2 and R3 repeats, with both repeats conferring binding specificity [48–51]. Pioneer work carried out on c-MYB from mouse lead to the identification of the MYB-core type I sequence (*CNGTT*) as one of the main targets of this class of TFs [52]. Further structural analyses have since then identified some conserved amino acid residues that are specifically involved in the interaction between the MYB DBDs and their DNA targets. For example, it has been demonstrated that Lys^40^ (K^40^) on the R2 helix 3 and Lys^43^/Asn^44^ (K^43^/N^44^) on the R3 helix 3 play a key role in the specific recognition of the *CNGTT* core sequence by specifically interacting with the first, third and last base pairs of this DNA sequence, respectively (amino acid position numbers are given in reference to Fig 5A; [49]). Additional studies have shown that the amino acid residues involved in the interaction between the MYB DBDs and their DNA target can vary accordingly to the considered target DNA sequence (S3 Fig). On this basis, the analysis of the binding specificity of the different DBDs as revealed by their BS prompted us to search for key features that could distinguish these various R2R3-MYB DBD subgroups.

For this purpose the amino acid sequences of the α-helix 3 of the R2 and R3 repeats (*i*.*e*. the ones involved in the interaction with the DNA residues) of each DBD subgroups were compiled and a consensus sequence LOGO generated (Fig 5A). It must be noted that some amino acids that are outside these helices might also affect the packing of the R2R3 repeats and by extension their binding specificities [53]. However, for the sake of simplicity in this study we concentrated on the DBD portions that directly participate in the interaction with DNA. In parallel, two DNA LOGOs corresponding to the consensus sequence of the two types of DNA motifs (group I and II) were generated. For this purpose DNA sequences that were displaying at least a 66% binding score with a given DBD subgroup were selected (Fig 5A).

The analysis of the protein sequence LOGOs showed that there is an overall good conservation between the 35 R2R3-MYB DBD subgroups, considering either the amino acid residues that are likely involved in the direct interaction with DNA or the surrounding ones (Fig 5A). Nevertheless, it emerges that α-helix 3 of the R2 repeat is more conserved than the one in R3. This observation suggests that helix 3 of the R3 repeat is most likely responsible for the differential interaction between the R2R3-MYBs and their DNA targets. Presumably these differences mostly affect the 3’-end of *cis*-elements, as illustrated on S4 Fig.

Seven DBD subgroups (corresponding to two clades; S2 Fig) displayed a low level of conservation of their amino acid sequence on helix 3 of both the R2 and R3 repeats when compared to the other subgroups (Fig 5A). First, the S21, S22, S23, S25 and SAt88 DBD clade appeared to be the most divergent as it was presenting amino acid variations all along helix 3 in both repeats. Interestingly, DBDs from this clade are more similar to the R1R2R3-MYB type found in animal and plants than to the other R2R3-MYB found in Arabidopsis, suggesting that these DBDs form a c-MYB-like clade (S2 Fig; [49, 50, 54]). Nevertheless this similarity does not apparently extend much further. R1R2R3-MYBs regulates cell cycle progression in both animal and plants [55, 56] whereas the R2R3-MYB proteins from this clade are mostly involved in plant specific processes [7, 8]. In this regard SAt88 appears to be an exception as AtMYB88 and AtMYB124/FLP (the two TFs in this subgroup) have been reported to control the expression of core cell cycle genes affecting cell proliferation in the stomatal lineage [15]. In contrast, subgroups SAt91 and SAt125 differ from all the other known MYB proteins in their third R3 alpha helices. Nevertheless, BLAST searches with SAt91 and SAt125 R3 α-helices showed their higher similarity with proteins from plant species than with other sequences.

DBD LOGOs analysis revealed also numerous subtle variations at the amino acid level that might be sufficient to modulate the interaction specificity of DBD subgroups with DNA motifs (Fig 5A). For example, SAt88 and SAt91 DBDs lack key amino acid residues involved in the interaction with DNA that are conserved in almost all plant and animal MYB proteins [50]. These include Lys^43^/Asn^44^ (K^43^/N^44^) replaced by Gly^43^/Lys^44^ (G^43^/K^44^) in SAt91 R3 alpha helixes 3, and Lys^40^ (K^40^) that is replaced by Arg^40^ (R^40^) in SAt88 R2 alpha helixes 3. Interestingly, B-MYB R2 α-helix 3 displays also an Arg^40^ placing the SAt88 R2 α-helix 3 in an intermediate position between c-MYB and B-MYB [49]. Furthermore, At88 DBDs are the only bearing Tyr^39^ (T^39^) and Arg^44^ (R^44^) in their R2 α-helixes 3. This later observation is quite surprising as in mammals [49] a Glu^44^ (E^44^) is found at this position whereas in plant it is generally a Leu^44^ (L^44^). This is even more surprising as the amino acid residue sitting at this position plays a conserved role in the interaction with the *GC* base pair found in the centre of the *CNGTT* core sequence (Fig 5A). Interestingly, if in the present study we have found that SAt88 R2R3-MYBs interact preferentially with the MYB-core type I (*TNGTT[A/G]*) *cis*-regulatory sequences (Fig 5A), previous work have demonstrated that AtMYB88 and AtMYB124/FLP have a strong affinity for the *[A/T/G][A/T/G]C[C/G][C/G]* consensus sequence [15]. This observation tends to indicate that the peculiar features observed for the R2 α-helixes 3 of SAt88 DBDs may play a central role in the specific DNA sequences recognition that are associated to this particular DBD subgroup. S19, S20a, S20b, SAt59 and SAt71 DBDs clade feature an Arg^47^ (R^47^) in R3 helix 3 unlike most other R2R3-MYBs, which have Asn^47^ (N^47^) on that site. Interestingly, an Asn^47^ to Arg^47^ substitution in MYB-Ph3 was associated *in vitro* with a shift in binding specificity from the MYB-core sequences toward the *AC*-elements [50]. However, it was also demonstrated that Ser^47^, Ile^47^ or His^47^ substitutions decreased overall MYB-Ph3 *in vitro* affinity without affecting its specificity, indicating that other residues are probably important for binding specificity [50]. S6 DBDs display an Arg^39^ (R^39^) and Ala^39^ (A^39^) on their R2 and R3 third helices, respectively. This specificity is sufficient to explain *in vivo* the target recognition differences between S5 (proanthocyanidin biosynthesis) and S6 (anthocyanin biosynthesis) DBDs [57]. Taken together, DBD LOGO analysis highlighted that subtle variations in the R2 and R3 helices 3 might be sufficient to modulate the interaction specificity of DBD subgroups with DNA motifs.

Considering the DNA targets, the analysis of the LOGOs generated from group I DNA sequences failed to clearly identify specific pattern or specificity for all DBDs (Fig 5A). This observation was indeed not surprising as this group of DNA motif was gathering DNA sequences that were strongly interacting in Y1H with most of the R2R3-MYBs that were assayed. Overall we observed that type I MYB-core consensus motif (*CNGTT[A/G]*) was the most represented, and that the *GTT*[*A*/*G*] motif was strongly conserved for all DBDs. No LOGO was generated for S24 DBD, reflecting its low BS for this group of DNA sequence (ranging from 25 to 50%; Fig 3).

In contrast, the analysis of LOGOs generated from group II DNA motifs revealed some specific features (Fig 5A). First, no LOGOs could be generated for seven DBDs indicating they preferentially target group I DNA sequences. Six additional DBD subgroups (*i*.*e*. S4, S5, S12, S18 and S25) were associated with a LOGO that corresponded to a unique DNA motif because of their overall low BS (below 66%; Fig 3). For most of the other DBDs we found a LOGO that was comparable to the group II consensus LOGO (*GNTAGTT[A/G]*), which is similar to the type II MYB-core consensus motif (*TNGTT[A/G]*). Three DBD subgroups associated with a LOGO corresponding to a unique *AC*-rich motif, namely S19, S24 and SAt46. If this finding was expected for S24 and SAt46, the absence of a similar result for S1, S2, S3, S13 and S24 DBDs was surprising (Fig 3). For this reason a second round of LOGO calculations was carried out for these five DBDs taking into consideration the sequences localised in between two consecutive DNA motifs (Fig 5B). As expected, LOGOs issued from this second analysis are closely related to the *AC*-rich DNA sequences. Interestingly, DBD subgroups that associates with *AC*-rich DNA sequences are scattered across most DBD clades except the two most divergent ones, c-MYB-like and At91/At125 clades (S3 Fig), indicating that this DNA-binding property has been acquire during the evolution of this family of TF. Surprisingly, the clade gathering DBDs from R2R3-MYBs whose activity depends on the formation of TTG1-dependent complexes [7, 39] seems to be excluded (S1 Fig), as if the formation of such protein complexes was unfavourable to this type of interaction.

## Discussion

Results gathered in the frame of this study support the idea that R2R3-MYB DNA binding plasticity and specificity have been acquired through the diversification of the DBDs during the course of the evolution of this class of plant-specific TFs. Indeed the concomitant evolution of the regulatory domain must have also played a role in this mechanism [3, 46]. Gene duplication, by expanding the number of R2R3-MYBs, has also increased the complexity level of this family of TF allowing functional redundancy for genes whose activity is central for plant growth and development (*e*.*g*. AtMYB88 and AtMYB124/FLP, that form DBD SAt88, which control stomata development and drought stress response; [58]). This important evolutionary process lead also to the neo-functionalization of duplicated R2R3-MYBs (*e*.*g*. AtMYB0/GL1 and AtMYB66/WER that control trichomes and root hairs development, respectively; [59]) as well as the acquisition of some specificity toward the control of gene expression in a time-, stress-, or tissue-specific manner (*e*.*g*. spatial regulation of flavonol biosynthesis by subgroup 7 R2R3-MYBs; [60]).

Genome-wide analysis (ChIP-seq or ChIP-chip) of complemented key R2R3-MYB loss-of-function mutants (*i*.*e*. representative of the R2R3-MYB and/or DBD subgroups) would undoubtedly be ideal in order to enhance our ability to understand and predict the interaction occurring between the R2R3-MYBs (or DBDs) and their DNA targets. Nevertheless, if this type of approach could generate crucial information notably on the dynamic and tissue specificity of these interactions, its set up remains technically demanding (when feasible), time consuming and its cost relatively expensive. An alternative would be to pursue the Y1H approach by extending the interaction matrix by increasing the number of assayed DNA motifs, ideally in a quantitative manner (*e*.*g*. Y1H experiments using *LacZ* as reporter gene), and to include the R2R3-MYBs that are specific to woody species (WPS, woody-preferential subgroups). Independently of the method used, this later point would allow determining if some DNA binding specificities have arose in perennial woody species [46]. Similarly, including R2R3-MYBs from non vascular plants (such the moss *Physcomitrella patens* or the single-cell green alga *Chlamydomonas reinhardtii*; http://planttfdb.cbi.pku.edu.cn/) would be valuable in order to evaluate how R2R3-MYB DNA binding specificities have evolved during the evolution of the plant lineage.

Identifying if some post-translational modifications (*e*.*g*. redox control, phosphatidic acid binding, phosphorylation, sumoylation, nitrosylation or ubiquitination) may influence the DNA binding capacity of each of the R2R3-MYB will remain one of the main challenges in elucidating the R2R3-MYB transcriptional regulatory code [11, 61–65]. Similarly, various studies indicate that the interaction between the R2R3-MYBs and some additional proteins is also a component of the R2R3-MYB transcriptional regulatory code as it affects their DNA binding capacity and as a consequence their transcriptional capacity. This is for example the case between AtMYB30 or AtMYB56 and BES1 (BRI1-EMS-SUPPRESSOR 1) in response to brassinosteroid signal [66, 67], or between the R2R3-MYBs belonging to subgroups 5, 6 and 15 (and closely related R2R3-MYBs) with the R/B-like bHLH TF and TTG1 for the transcriptional control of cell patterning (trichomes and roots hair) or flavonoid (anthocyanins and proanthocyanidins) and mucilage biosynthesis [39, 68, 69]. This is also the case for AtMYB91/AS1 (ASYMETRIC LEAVES 1) that interacts with AS2 (a LOB domain protein) in order to repress the expression of KNOX genes that ultimately induces determinate lateral organs formation [70].

In conclusion, this study provides a comprehensive *in vivo* analysis of R2R3-MYB binding activities that should help in predicting new DNA motifs and identifying new putative target genes for each member of this very large family of TFs. In a broader perspective, the generated data will help to better understand how TF interact with their target DNA sequences and provide new information that may be useful for biotechnological engineering of various plant traits.

## Materials and Methods

### Cloning of R2R3-MYB transcription factors coding sequence (cds)

A mix of cDNA from all parts and developmental phases of *Arabidopsis thaliana* (Columbia) plants was used as a template to amplify (Phusion DNA polymerase, GC buffer, Thermo Scientific–Finnzymes) the open reading frame (ORF) of the R2R3-MYB transcription factors. Nested PCR with appropriate oligonucleotides (see the cloning strategy at http://www-urgv.versailles.inra.fr/atome/) was used to amplify ORFs with or without stop codons flanked by the Gateway® *attB1* and *attB2* sites (Invitrogen). It must be noted that 5’-end primer contained the Shine-Dalgarno and Kozak sequences (in between the Gateway® *attB1* site and the *ATG*) in order to improve the translation of the cloned R2R3-MYB in bacteria or eukaryotic cells, respectively. The fragments obtained were then BP recombined into the pDONR207 vector. When the amplification of an ORF was unsuccessful the exons were amplified separately using genomic DNA as template. The exon fragments were then fused by PCR in order to reproduce the ORF sequence that is described at the TAIR database (http://www.arabidopsis.org; TAIR 9). Primers (from Eurofins MWG Operon) are listed S5 Table. pDONR207 constructs were sequenced to ensure ORFs integrity. From the pDONR207 vectors containing the ORFs an ordered library has been build. Examples of ORFs functional validation are given S6 Fig.

### Yeast one-hybrid (Y1H) experiments

We have used the ligation independent cloning system (LIC; [71]) to clone 16 different known *cis*-regulatory sequences [8, 14, 16, 17, 23–27, 72] into the pHISi yeast one-hybrid vector (Clontech). pHISi constructs were sequenced to ensure their integrity. The pHis-LIC construct containing the *cis*-element was then stably transformed into yeast (*Saccharomyces cerevisiae*, EGY48 α-type mating strain) at the *URA3* locus [73]. The ordered transcription factor library (pDONR207) containing stop codons was LR recombined into pDEST22 vector that allows fusion with the GAL4-activating domain and was then transformed into the yeast a-type mating strain YM4271. Prior yeast transformation, pDEST22 constructs were sequenced to ensure ORF integrity. Mating, diploid cell selection and interaction determination was carried out as described in [22]. Y1H data for all R2R3-MYBs have been compiled in footprintDB database (http://floresta.eead.csic.es/footprintdb/index.php; [74].

### Precision, recall and false discovery rate calculations

Precision (P, estimate the proportion of true positive), recall (R, estimate the true positive rate) and false discovery rate (FDR) calculations associated with our Y1H dataset were based on published positive and negative interactions (S1, S2 and S4 Tables). P = TP / (TP+FP), R = TP / (TP+FN), and FDR = FP / (TP+FP). TP: true positive (confirmation in our dataset of published data), FP: false positive (interaction identified in our dataset that was published as not occurring), FN: false negative (published interaction that was not confirmed in our dataset).

### Bioinformatics

#### Phylogenetic tree and binding matrix

R2R3-MYB protein sequences were aligned with clustal-omega [47] and only the block corresponding to R2R3 domains exactly as defined by [3] was conserved for further analysis. Then the LG substitution model with fixed gamma and invariant rates was used to build a maximum likelihood phylogeny with phylogeny.fr [75]. The tree was then edited and branches collapsed with FigTree (http://tree.bio.ed.ac.uk/software/figtree). The resulting subgroups were named trying to maximize the overlap with previous MYB classifications. R library corrplot was used to plot a matrix of mean binding affinities inferred in the Y1H assays.

#### Sequence logos

LOGOs of MYB recognition α-helices of protein members of a subgroup were generated with WebLogo [76]. For the target *cis*-elements, those bound with affinity ‘++’ by at least two thirds of a subgroup were used as input for WebLogo aligned as in Fig 1.

#### Protein-DNA interfaces

The interfaces of Protein Data Bank entries 1MSE [49] and 2KDZ [77], as dissected by 3d-footprint [78], were aligned with tfcompare [79].

#### Mutual information calculation

Columns in multiple alignments of recognition helices and also in multiply aligned *cis*-elements of clusters of subgroups in S5 Fig were used to calculate first the frequency of independent occurrence of individual amino acids and DNA bases and then their co-occurrence frequency. Mutual information was then calculated with the standard equation with ln as logarithmic function.

## Supporting Information

S1 Fig Binding specificity of selected R2R3-MYB subgroups. Heat map representation of the Y1H results observed with the most discriminating DNA motifs (*i*.*e*. interacting with a small set of R2R3-MYB). In this representation are only considered R2R3-MYBs subgroups for which at least 75% of the protein members display a positive or negative interaction with DNA motifs from group (**A**) IIa, (**B**) IIb, (**C**) IIc and (**D**) IId. Red stars indicate R2R2-MYBs displaying a preferential affinity toward *AC*-rich DNA sequences. Black stars indicate R2R2-MYBs involved in TTG1-depedent complexes [37].
(PDF)

S2 Fig Maximum Likelihood phylogenetic tree constructed using the R2R3-MYB DNA binding domains (DBDs). In red: DBD subgroups that match previously described R2R3-MYB subgroups [3, 7], in green: DBD subgroups that correspond to split previously described R2R3-MYB subgroups, in blue: DBD subgroups that were not corresponding to any previously described R2R3-MYB subgroups (ungrouped R2R3-MYBs) in *Arabidopsis thaliana*. Pink arrows: DBD subgroups that display a strong *trans*-activation affinity toward the *AC*-rich DNA sequences.
(PDF)

S3 Fig Examples of protein-DNA interfaces captured in protein data bank (PDB) structures of MYB transcription factors. Numbers inside nitrogen bases indicate the number of contacts with protein-side chains within 4.5 Å. Dashed bases correspond to the core *GTT* motif, which is recognized by conserved amino acid side-chains in entries 1MSE [49] and 2KDZ [77]. Upstream bases are also specifically recognized but the involved side-chains are not conserved. Filled bases display DNA geometry alterations typical of indirect readout mechanisms. The horizontal double head arrows below delimitate the segments where recognition helices make direct contacts with DNA nitrogen bases as seen in these and on another PDB entries. Numbers refers to the amino acid positions from the start codon (Met) which correspond to the following positions in Fig 5A: Glu^132^ (1MSE) and Glu^41^ (2KDZ) = Glu^44^/Leu^44^ (*A*. *thaliana* R2 helix 3), Asn^179^ (1MSE) and Asn^88^ (2KDZ) = Asn^40^ (*A*. *thaliana* R3 helix 3), and Asn^183^ (1MSE) and Asn^92^ (2KDZ) = Asn^44^ (*A*. *thaliana* R2 helix 3). These structures show that when comparing the DBD/DNA interface of two MYB proteins that interact with two different types of MYB-core motif (*i*.*e*. type I in the top part *vs* type II in the bottom part) it can be observed that indeed some amino acids are conserved. This is for instance the case for two residues that recognized the *GTT* DNA motif, namely the Glu^44^ and the Asn^40^ from the R2 and R3 helices 3, respectively. It is noteworthy that the Glu^44^ residue is generally replaced in plants by a Leu^44^. However, it can also be observed that the number of amino acid residues (and as a consequence the type) that directly interact with each of the nucleotides surrounding the *GTT* core is highly variable.
(PDF)

S4 Fig Relationship between interface protein residues and positions of the corresponding R2R3-MYB target elements as calculated for three (A, B, C) clusters of neighbor subgroups as seen in Fig 5. Arrows connect bases to residues displaying maximum mutual information (in bits) calculated between columns of multiply aligned recognition helices and columns of aligned *cis*-elements. Dashed lines mark residues that are not directly contacting DNA but affect helix packing.
(PDF)

S5 Fig MYB21 SELEX (systematic evolution of ligands by exponential enrichment) experiment. *MYB21* cDNA was cloned into the expression vector pDEST17 (Invitrogen), the MYB21 recombinant protein was produced in *Escherichia coli* (BL21) and purified using the Ni-NTA agarose kit (Qiagen) according to manufacturer recommendations. PCR SELEX was carried out as follow: purified MYB21 was incubated with a mix of random primers (5’-TCGACTCGAGTCGACATCGNNNNNNNNNNNNNNNNNNGGATCCTGCAAATTCGCG-3’) and immuno-precipitated (IP) with nProtein A Sepharose™ 4 Fast Flow (GE healthcare) according to manufacturers instructions using the low salt buffer and Anti-His 6 -Peroxidase (Roche Life Science). DNA fragments were amplified following IP with the following forward and reverse primers: 5’-CGCGAATTTGCAGGATCC-3’ and 5’-TCGACTCGAGTCGACATCG-3’. After six cycles of PCR SELEX, DNA fragments were cloned and sequenced. (**A**) Sequence alignment of 22 DNA fragments bound to MYB21 identified after sequencing. Aligned sequences are centred on the consensus MYB core DNA motif, *[C/T]NGTT[A/G]*. (**B**) Logo generated from the 21 DNA motifs. (**C**) Logo generated from the DNA motifs that were similar to the MYB-core type I sequence: *CNGTT[A/G]*. (**D**) Logo generated from the DNA motifs that were similar to the MYB-core type II sequence: *TNGTT[A/G]*.
(PDF)

S6 Fig Examples of functional characterization of cloned R2R3-MYB open reading frames (ORFs). (**A**) Transient expression assays: green fluorescent protein (GFP) intensity was measured in *Physcomitrella patens* protoplasts co-transfected with *proBAN76*:*35Smini*:*GFP* alone (negative control) or together with AtTT8, AtTTG1 and AtTT2. TT2 is a key transcriptional regulator of proanthocyanidin (PA) biosynthesis (flavonoid) in seeds. TT2, together with TT8/bHLH042 and TTG1 (TRANSPARENT TESTA GLABRA 1, a WD repeat containing protein) form a ternary protein complex that specifically regulates the expression of genes involved in this pathway, such as *BANYULS*. The origin of AtTT2 being from either a previous study (grey bars, [27]) that corresponds to the positive control or cloned within the frame of this study (black bars). Error bars ± SE. *t*-test significance: ***, P < 0.001. none: promoter alone (**B-C**) *Arabidopsis thaliana* mutant complementation experiments: (**B**) *myb5 tt2 transparent testa* phenotype (*i*.*e*. yellow seeds deprived of PAs) was complemented by expressing *TT2/AtMYB123* or *AtMYB5* under the control of the *TT8* promoter as previously described [41], (**C**) *gl1-1* lack of trichomes was reverted by overexpressing *GL1* (*GLABRA1/AtMYB0*) and *WER* (*WEREWOLF/AtMYB66*) ORFs, encoding functional homologues.
(PDF)

S1 Table Previously described interactions between R2R3-MYBs and consensus DNA motifs in *Arabidopsis thaliana*. (PDF)

S2 Table Previously described interactions between R2R3-MYBs and target DNA sequences in *Arabidopsis thaliana*. (PDF)

S3 Table DNA motifs analysed in this study. (PDF)

S4 Table Summary of yeast one-hybrid screens results. (PDF)

S5 Table Primers used in this study. (PDF)
