# Supplementary material for: Analysis of the DNA-Binding Activities of the Arabidopsis R2R3-MYB Transcription Factor Family by One-Hybrid Experiments in Yeast
Source: PLoS One. 2015 Oct 20;10(10):e0141044. doi: 10.1371/journal.pone.0141044 (PMC4613820; doi:10.1371/journal.pone.0141044)
Supplement: S1 Fig — Heat map representation of the Y1H results observed with the most discriminating DNA motifs (i.e. interacting with a small set of R2R3-MYB). In this representation are only considered R2R3-MYBs subgroups for which at least 75% of the protein members display a positive or negative interaction with DNA motifs from group (A) IIa, (B) IIb, (C) IIc and (D) IId. Red stars indicate R2R2-MYBs displaying a preferential affinity toward AC-rich DNA sequences. Black stars indicate R2R2-MYBs involved in TTG1-depedent complexes [37]. (PDF) [file pone.0141044.s001.pdf]

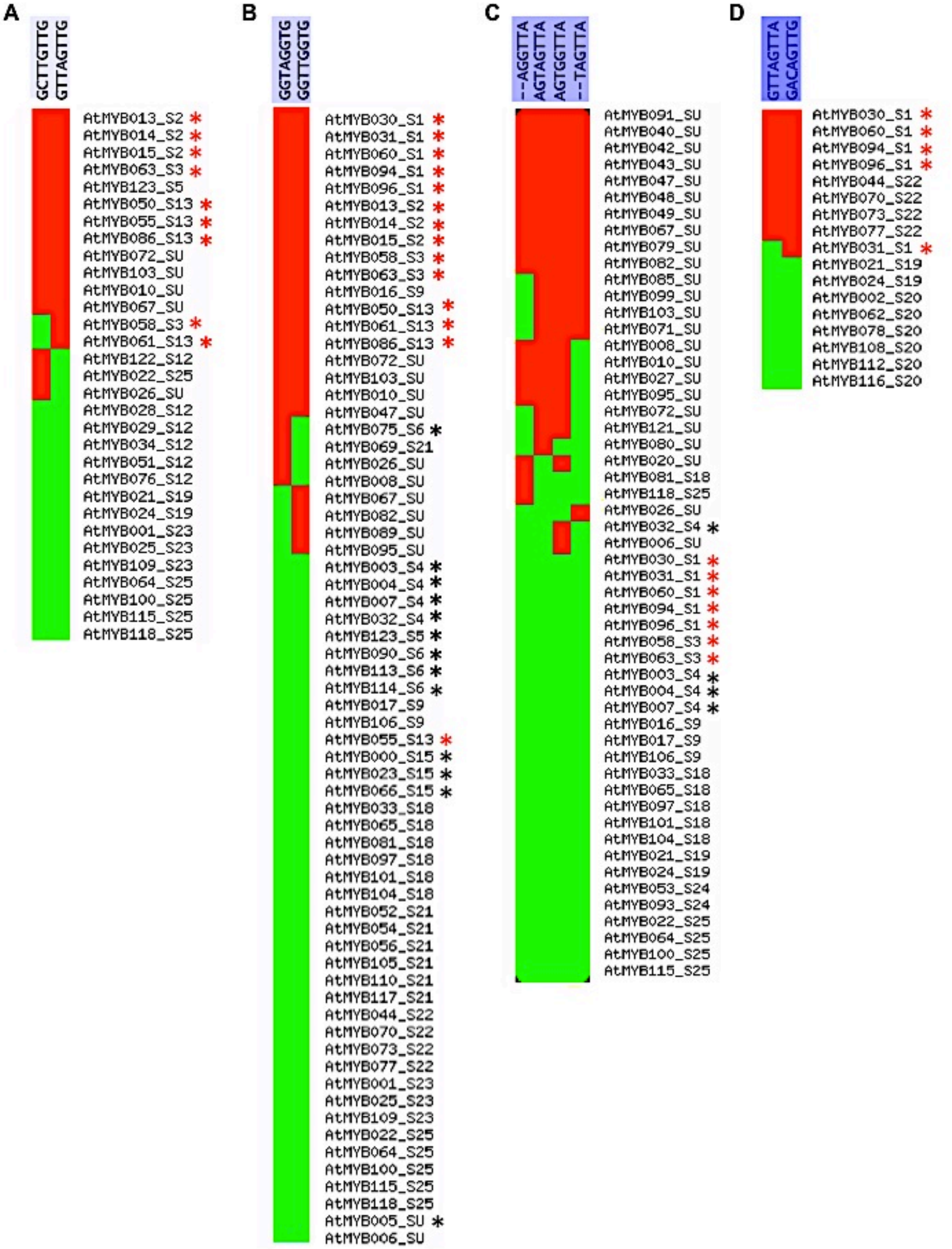

Figure S1. Binding specificity of selected R2R3-MYB subgroups. Heat map representation of the Y1H results observed with the most discriminating DNA motifs (*i.e.* interacting with a small set of R2R3-MYB). In this representation are only considered R2R3-MYBs subgroups for which at least 75% of the protein members display a positive or negative interaction with DNA motifs from group (**A**) IIa, (**B**) IIb, (**C**) IIc and (**D**) IId. Red stars indicate R2R2-MYBs displaying a preferential affinity toward *AC*-rich DNA sequences. Black stars indicate R2R2-MYBs involved in TTG1-depedent complexes (Zimmermann et ., 2004).
