# Supplementary material for: Analysis of the DNA-Binding Activities of the Arabidopsis R2R3-MYB Transcription Factor Family by One-Hybrid Experiments in Yeast
Source: PLoS One. 2015 Oct 20;10(10):e0141044. doi: 10.1371/journal.pone.0141044 (PMC4613820; doi:10.1371/journal.pone.0141044)
Supplement: S2 Fig — In red: DBD subgroups that match previously described R2R3-MYB subgroups [3, 7], in green: DBD subgroups that correspond to split previously described R2R3-MYB subgroups, in blue: DBD subgroups that were not corresponding to any previously described R2R3-MYB subgroups (ungrouped R2R3-MYBs) in Arabidopsis thaliana. Pink arrows: DBD subgroups that display a strong trans-activation affinity toward the AC-rich DNA sequences. (PDF) [file pone.0141044.s002.pdf]

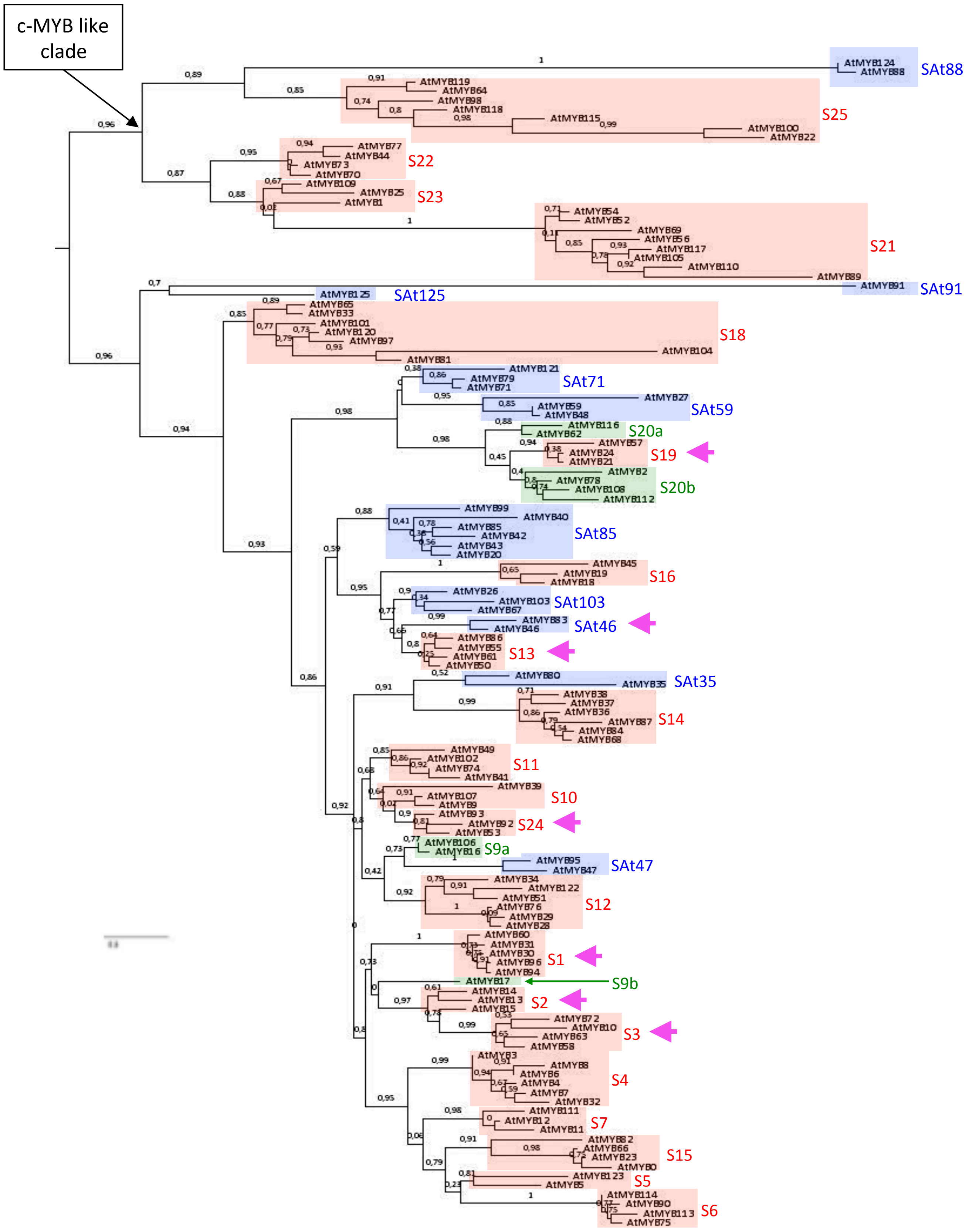

Figure S2. Maximum Likelihood phylogenetic tree constructed using the R2R3-MYB DNA binding domains (DBDs). In red: DBD subgroups that match previously described R2R3-MYB subgroups (Dubos et al., 2010; Stracke et al., 2001), in green: DBD subgroups that correspond to split previously described R2R3-MYB subgroups, in blue: DBD subgroups that were not corresponding to any previously described R2R3-MYB subgroups (ungrouped R2R3-MYBs) in *Arabidopsis thaliana*. Pink arrows: DBD subgroups that display a strong *trans*-activation affinity toward the *AC*-rich DNA sequences.
