# Supplementary material for: Analysis of the DNA-Binding Activities of the Arabidopsis R2R3-MYB Transcription Factor Family by One-Hybrid Experiments in Yeast
Source: PLoS One. 2015 Oct 20;10(10):e0141044. doi: 10.1371/journal.pone.0141044 (PMC4613820; doi:10.1371/journal.pone.0141044)
Supplement: S3 Fig — Numbers inside nitrogen bases indicate the number of contacts with protein-side chains within 4.5 Å. Dashed bases correspond to the core GTT motif, which is recognized by conserved amino acid side-chains in entries 1MSE [49] and 2KDZ [77]. Upstream bases are also specifically recognized but the involved side-chains are not conserved. Filled bases display DNA geometry alterations typical of indirect readout mechanisms. The horizontal double head arrows below delimitate the segments where recognition helices make direct contacts with DNA nitrogen bases as seen in these and on another PDB entries. Numbers refers to the amino acid positions from the start codon (Met) which correspond to the following positions in Fig 5A: Glu132 (1MSE) and Glu41 (2KDZ) = Glu44/Leu44 (A. thaliana R2 helix 3), Asn179 (1MSE) and Asn88 (2KDZ) = Asn40 (A. thaliana R3 helix 3), and Asn183 (1MSE) and Asn92 (2KDZ) = Asn44 (A. thaliana R2 helix 3). These structures show that when comparing the DBD/DNA interface of two MYB proteins that interact with two different types of MYB-core motif (i.e. type I in the top part vs type II in the bottom part) it can be observed that indeed some amino acids are conserved. This is for instance the case for two residues that recognized the GTT DNA motif, namely the Glu44 and the Asn40 from the R2 and R3 helices 3, respectively. It is noteworthy that the Glu44 residue is generally replaced in plants by a Leu44. However, it can also be observed that the number of amino acid residues (and as a consequence the type) that directly interact with each of the nucleotides surrounding the GTT core is highly variable. (PDF) [file pone.0141044.s003.pdf]

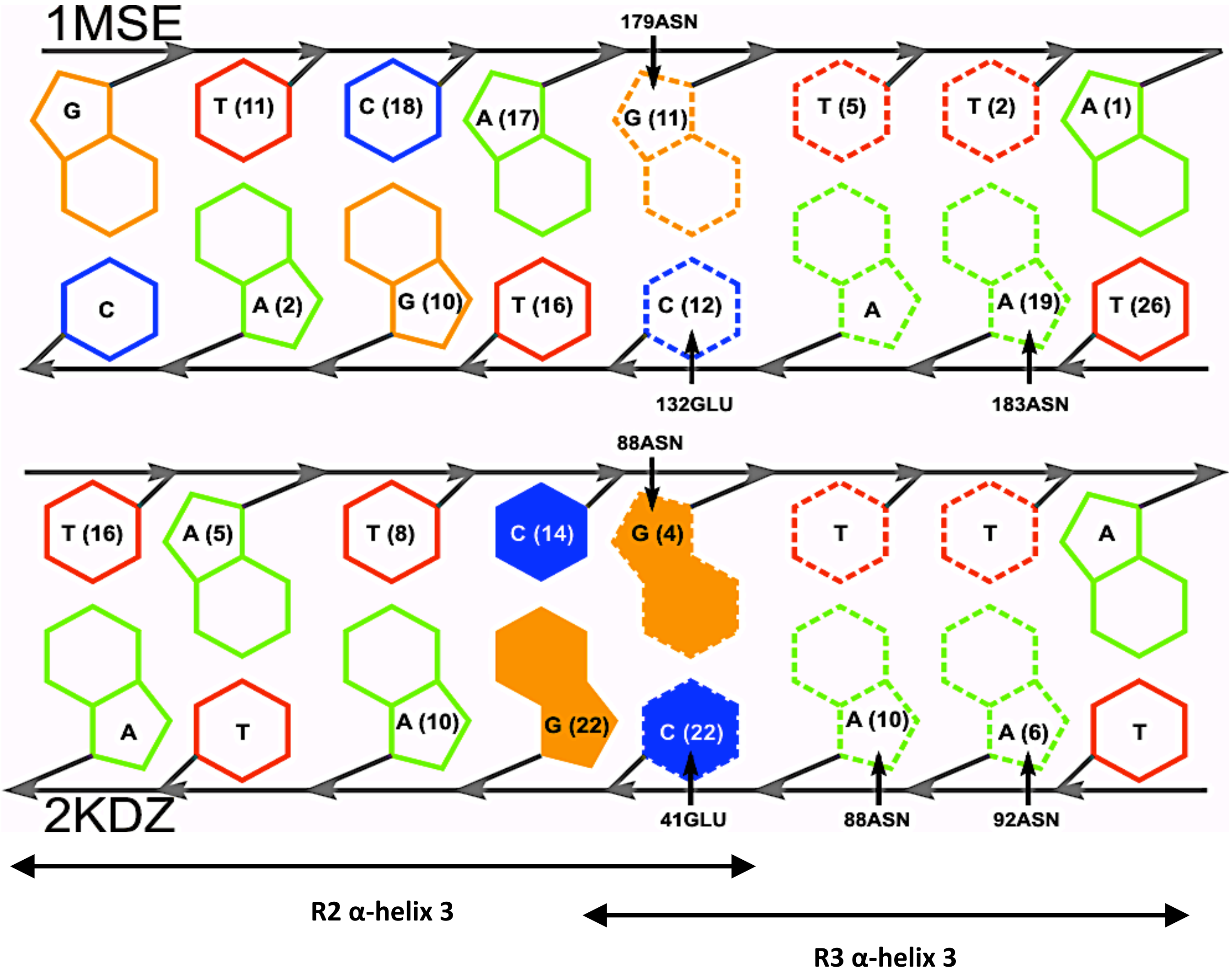

Figure S3. Examples of protein-DNA interfaces captured in protein data bank (PDB) structures of MYB transcription factors. Numbers inside nitrogen bases indicate the number of contacts with protein-side chains within 4.5 Å. Dashed bases correspond to the core *GTT* motif, which is recognized by conserved amino acid side-chains in entries 1MSE (Ogata *et al*., 1994) and 2KDZ (Lou *et al*., 2009). Upstream bases are also specifically recognized but the involved side-chains are not conserved. Filled bases display DNA geometry alterations typical of indirect readout mechanisms. The horizontal double head arrows below delimitate the segments where recognition helices make direct contacts with DNA nitrogen bases as seen in these and on another PDB entries. Numbers refers to the amino acid positions from the start codon (Met) which correspond to the following positions in Figure 5A: Glu^{132} (1MSE) and Glu^{41} (2KDZ) = Glu^{44}/Leu^{44} (*A. thaliana* R2 helix 3), Asn^{179} (1MSE) and Asn^{88} (2KDZ) = Asn^{40} (*A. thaliana* R3 helix 3), and Asn^{183} (1MSE) and Asn^{92} (2KDZ) = Asn^{44} (*A. thaliana* R2 helix 3). These structures show that when comparing the DBD/DNA interface of two MYB proteins that interact with two different types of MYB-core motif (*i.e.* type I in the top part *vs* type II in the bottom part) it can be observed that indeed some amino acids are conserved. This is for instance the case for two residues that recognized the *GTT* DNA motif, namely the Glu^{44} and the Asn^{40} from the R2 and R3 helices 3, respectively. It is noteworthy that the Glu^{44} residue is generally replaced in plants by a Leu^{44}. However, it can also be observed that the number of amino acid residues (and as a consequence the type) that directly interact with each of the nucleotides surrounding the *GTT* core is highly variable.
