# Supplementary material for: Analysis of the DNA-Binding Activities of the Arabidopsis R2R3-MYB Transcription Factor Family by One-Hybrid Experiments in Yeast
Source: PLoS One. 2015 Oct 20;10(10):e0141044. doi: 10.1371/journal.pone.0141044 (PMC4613820; doi:10.1371/journal.pone.0141044)
Supplement: S4 Fig — Arrows connect bases to residues displaying maximum mutual information (in bits) calculated between columns of multiply aligned recognition helices and columns of aligned cis-elements. Dashed lines mark residues that are not directly contacting DNA but affect helix packing. (PDF) [file pone.0141044.s004.pdf]

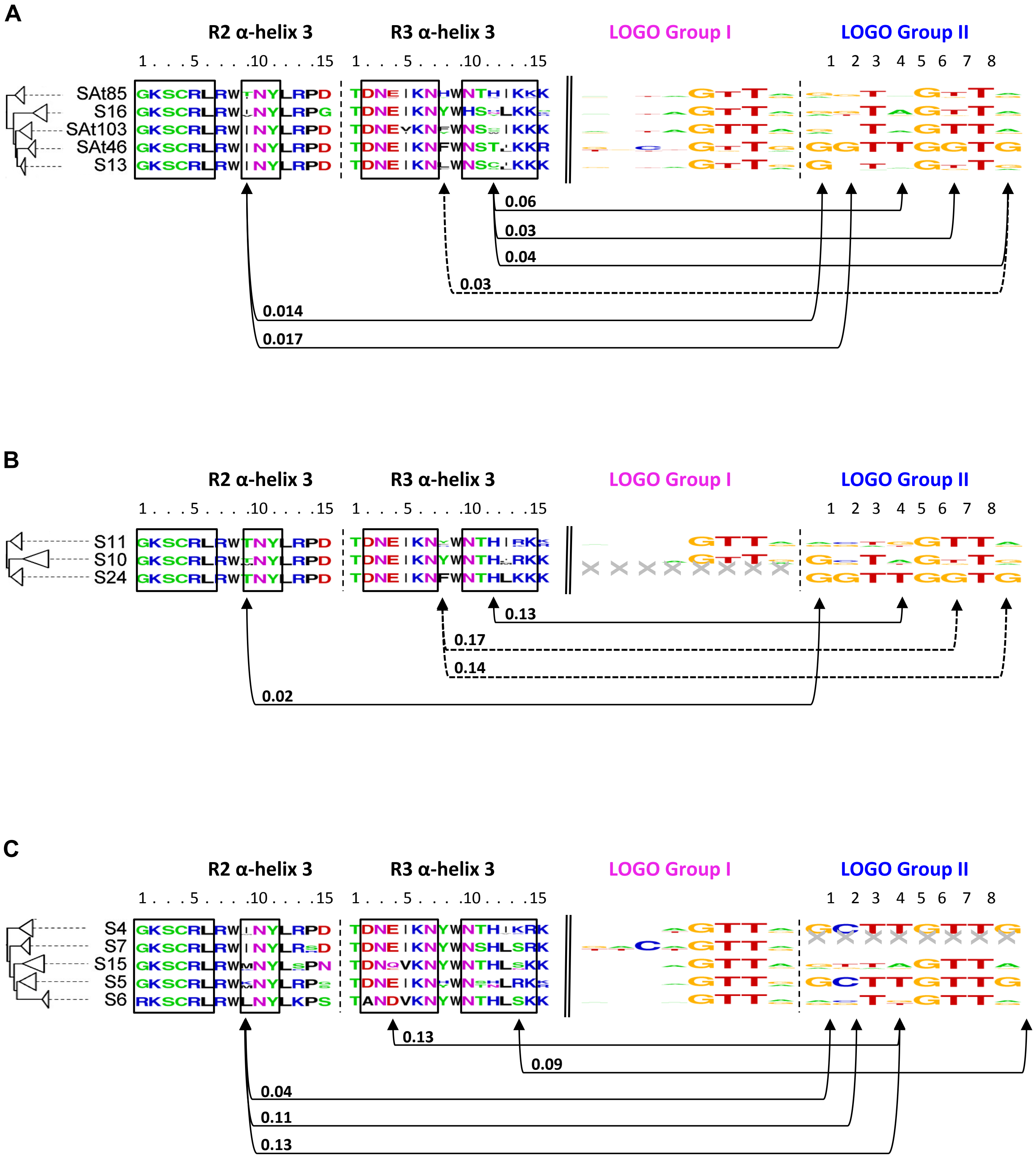


**Figure S4. Relationship between interface protein residues and positions of the corresponding R2R3-MYB target elements as calculated for three (A, B, C) clusters of neighbor subgroups as seen in Figure 5.** Arrows connect bases to residues displaying maximum mutual information (in bits) calculated between columns of multiply aligned recognition helices and columns of aligned *cis*-elements. Dashed lines mark residues that are not directly contacting DNA but affect helix packing.
