# Supplementary material for: Analysis of the DNA-Binding Activities of the Arabidopsis R2R3-MYB Transcription Factor Family by One-Hybrid Experiments in Yeast
Source: PLoS One. 2015 Oct 20;10(10):e0141044. doi: 10.1371/journal.pone.0141044 (PMC4613820; doi:10.1371/journal.pone.0141044)
Supplement: S5 Fig — MYB21 cDNA was cloned into the expression vector pDEST17 (Invitrogen), the MYB21 recombinant protein was produced in Escherichia coli (BL21) and purified using the Ni-NTA agarose kit (Qiagen) according to manufacturer recommendations. PCR SELEX was carried out as follow: purified MYB21 was incubated with a mix of random primers (5’-TCGACTCGAGTCGACATCGNNNNNNNNNNNNNNNNNNGGATCCTGCAAATTCGCG-3’) and immuno-precipitated (IP) with nProtein A Sepharose™ 4 Fast Flow (GE healthcare) according to manufacturers instructions using the low salt buffer and Anti-His 6 -Peroxidase (Roche Life Science). DNA fragments were amplified following IP with the following forward and reverse primers: 5’-CGCGAATTTGCAGGATCC-3’ and 5’-TCGACTCGAGTCGACATCG-3’. After six cycles of PCR SELEX, DNA fragments were cloned and sequenced. (A) Sequence alignment of 22 DNA fragments bound to MYB21 identified after sequencing. Aligned sequences are centred on the consensus MYB core DNA motif, [C/T]NGTT[A/G]. (B) Logo generated from the 21 DNA motifs. (C) Logo generated from the DNA motifs that were similar to the MYB-core type I sequence: CNGTT[A/G]. (D) Logo generated from the DNA motifs that were similar to the MYB-core type II sequence: TNGTT[A/G]. (PDF) [file pone.0141044.s005.pdf]

A

```
>NB9              GGTGCCACAGTTCCGCC
>NB11            GGGGTGCAGTGTTTCTGG
>NB27     GGCGGGATGTGTGGTTTG
>NB33                       CCGGTTGCGCTACGGCGG
>NB34        GGGCACAGACCATTCTCC
>NB37                GGGGGGGGTTCATGCGTG
>NB39                          TGTTCTCCGACCTCCGGG
>NB17     GCCGGATCGGTGTGTTGC
>NB40      GCAATATGGTCGGTT
>NB2       GGGCCATGCGCGGTTCC
>NB4       GGGACCTGACCCGTTTCC
>NB7              GGAGGTCCTCTTGCGTGT
>NB1                CCTCCGCGGTAAGCGACC
>NB15                     TGTCTGTACCGGCGG
>NB16               GGCGAGCATTTAGGTGCC
>NB22                   CCCATCGTCACCCCCCGG
>NB23             GACCATATGGTGGGCTGG
>NB12                        CACAGTAAGACCGCTAC
>NB28        CGGGACAAGTCGTTAGGG
>NB8            GGGGAGAGCGATTAGCCC
>NB24               GGCCGTCCGTTACGTGTG
>NB21   GGGGGGCCTGACTCGTT
```

B C D

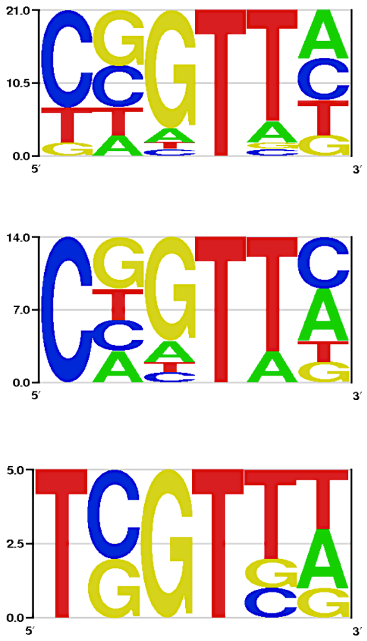

Figure S5: MYB21 SELEX (systematic evolution of ligands by exponential enrichment) experiment. *MYB21* cDNA was cloned into the expression vector pDEST17 (Invitrogen), the MYB21 recombinant protein was produced in *Escherichia coli* (BL21) and purified using the Ni-NTA agarose kit (Qiagen) according to manufacturer recommendations. PCR SELEX was carried out as follow: purified MYB21 was incubated with a mix of random primers (5'-TCGACTCGAGTCGACATCGNNNNNNNNNNNNNNNNNNGGATCCTGCAAATTCGCG-3') and immuno-precipitated (IP) with nProtein A Sepharose™ 4 Fast Flow (GE healthcare) according to manufacturers instructions using the low salt buffer and Anti-His 6 -Peroxidase (Roche Life Science). DNA fragments were amplified following IP with the following forward and reverse primers: 5'-CGCGAATTTGCAGGATCC-3' and 5'-TCGACTCGAGTCGACATCG-3'. After six cycles of PCR SELEX, DNA fragments were cloned and sequenced. (**A**) Sequence alignment of 22 DNA fragments bound to MYB21 identified after sequencing. Aligned sequences are centred on the consensus MYB core DNA motif, *[C/T]NGTT[A/G]*. (**B**) Logo generated from the 21 DNA motifs. (**C**) Logo generated from the DNA motifs that were similar to the MYB-core type I sequence: *CNGTT[A/G]*. (**D**) Logo generated from the DNA motifs that were similar to the MYB-core type II sequence: *TNGTT[A/G]*.
