# Supplementary material for: Analysis of the DNA-Binding Activities of the Arabidopsis R2R3-MYB Transcription Factor Family by One-Hybrid Experiments in Yeast
Source: PLoS One. 2015 Oct 20;10(10):e0141044. doi: 10.1371/journal.pone.0141044 (PMC4613820; doi:10.1371/journal.pone.0141044)
Supplement: S6 Fig — (A) Transient expression assays: green fluorescent protein (GFP) intensity was measured in Physcomitrella patens protoplasts co-transfected with proBAN76:35Smini:GFP alone (negative control) or together with AtTT8, AtTTG1 and AtTT2. TT2 is a key transcriptional regulator of proanthocyanidin (PA) biosynthesis (flavonoid) in seeds. TT2, together with TT8/bHLH042 and TTG1 (TRANSPARENT TESTA GLABRA 1, a WD repeat containing protein) form a ternary protein complex that specifically regulates the expression of genes involved in this pathway, such as BANYULS. The origin of AtTT2 being from either a previous study (grey bars, [27]) that corresponds to the positive control or cloned within the frame of this study (black bars). Error bars ± SE. t-test significance: ***, P < 0.001. none: promoter alone (B-C) Arabidopsis thaliana mutant complementation experiments: (B) myb5 tt2 transparent testa phenotype (i.e. yellow seeds deprived of PAs) was complemented by expressing TT2/AtMYB123 or AtMYB5 under the control of the TT8 promoter as previously described [41], (C) gl1-1 lack of trichomes was reverted by overexpressing GL1 (GLABRA1/AtMYB0) and WER (WEREWOLF/AtMYB66) ORFs, encoding functional homologues. (PDF) [file pone.0141044.s006.pdf]

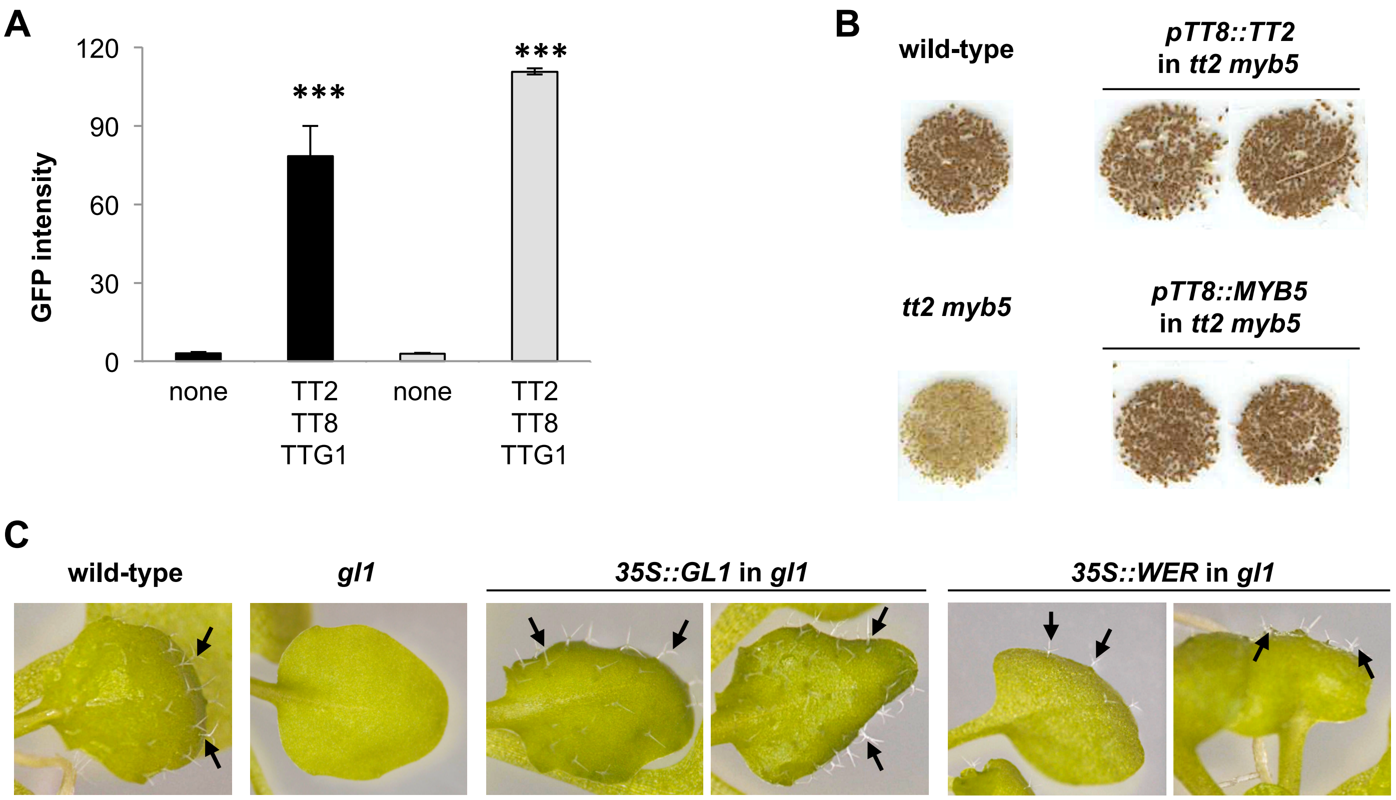

Figure S6: Examples of functional characterization of cloned R2R3-MYB open reading frames (ORFs). (**A**) Transient expression assays: green fluorescent protein (GFP) intensity was measured in *Physcomitrella patens* protoplasts co-transfected with *proBAN76:35Smini:GFP* alone (negative control) or together with AtTT8, AtTTG1 and AtTT2. TT2 is a key transcriptional regulator of proanthocyanidin (PA) biosynthesis (flavonoid) in seeds. TT2, together with TT8/bHLH042 and TTG1 (TRANSPARENT TESTA GLABRA 1, a WD repeat containing protein) form a ternary protein complex that specifically regulates the expression of genes involved in this pathway, such as *BANYULS*. The origin of AtTT2 being from either a previous study (grey bars, Thévenin *et al*., 2012) that corresponds to the positive control or cloned within the frame of this study (black bars). Error bars ± SE. *t*-test significance: ***, $P < 0.001$. none: promoter alone (**B-C**) *Arabidopsis thaliana* mutant complementation experiments: (**B**) *myb5 tt2 transparent testa* phenotype (*i.e.* yellow seeds deprived of PAs) was complemented by expressing *TT2/AtMYB123* or *AtMYB5* under the control of the *TT8* promoter as previously described (Xu *et al*., 2013), (**C**) *gl1-1* lack of trichomes was reverted by overexpressing *GL1* (*GLABRA1/AtMYB0*) and *WER* (*WEREWOLF/AtMYB66*) ORFs (encoding functional homologues) as previously described (Lee and Schiefelbein, 2001).
