# Supplementary material for: Analysis of the DNA-Binding Activities of the Arabidopsis R2R3-MYB Transcription Factor Family by One-Hybrid Experiments in Yeast
Source: PLoS One. 2015 Oct 20;10(10):e0141044. doi: 10.1371/journal.pone.0141044 (PMC4613820; doi:10.1371/journal.pone.0141044)
Supplement: S1 Table — (PDF) [file pone.0141044.s007.pdf]

S1 Table. Summary of previously described interactions between R2R3-MYBs and consensus DNA motifs in Arabidopsis thaliana. MYB-core type I: CNGTTR (R=A/G), MYB-core type II: TNGTTR (R=A/G), AC-element ACCWAMY (W=A/T, M=A/C, and Y=T/C) and FLP/MYB88 binding sites: DDCSS (D=A/T/G, and S=C/G). MYB-core like consensus sequences refers to sequences similar to either MYB-core type I or type II. Details are given Table S2.

Subgroup	R2R3-MYB	Consensus DNA motif
**S1**	AtMYB030	MYB-core type II
**S1**	AtMYB031 / ATY13	*AC*-element
**S1**	AtMYB060	*AC*-element
**S1**	AtMYB094	*AC*-element
**S1**	AtMYB096	MYB-core type I and MYB-core type II
**S2**	AtMYB013	MYB-core type I and MYB-core type II
**S2**	AtMYB015	*AC*-element
**S3**	AtMYB058	*AC*-element
**S5**	AtMYB123 / TT2	MYB-core type I and MYB-core type II
**S6**	AtMYB075 / PAP1	*AC*-element
**S7**	AtMYB111 / PFG3	*AC*-element
**S10**	AtMYB107	*AC*-element
**S11**	AtMYB102 / AtM4	*AC*-element
**S13**	AtMYB050	*AC*-element
**S13**	AtMYB055	*AC*-element
**S13**	AtMYB061	*AC*-element
**S13**	AtMYB086	*AC*-element
**S14**	AtMYB084	*AC*-element
**S15**	AtMYB000 / GL1	MYB-core type II
**S15**	AtMYB023	MYB-core type II and AC-element
**S15**	AtMYB066 / WER	MYB-core type I, MYB-core type II and AC-element
**S16**	AtMYB019	*AC*-element
**S16**	AtMYB045	*AC*-element
**S18**	AtMYB033	*AC*-element
**S18**	AtMYB081	*AC*-element
**S19**	AtMYB021	MYB-core type I, MYB-core type II and MYB-core Like
**S20**	AtMYB002	MYB-core type I and AC-element
**S20**	AtMYB108 / BOS1	*AC*-element
**S20**	AtMYB112	*AC*-element
**S21**	AtMYB052	MYB-core type I and MYB-core type II
**S22**	AtMYB044 / AtMYBR1	MYB-core type I, MYB-core type II and MYB-core Like
**S22**	AtMYB073	*AC*-element
**S22**	AtMYB077 / AtMYBR2	MYB-core type I, MYB-core type II and MYB-core Like
**S24**	AtMYB092	*AC*-element
**S25**	AtMYB118	MYB-core type I
	AtMYB046	*AC*-element
	AtMYB049	*AC*-element
	AtMYB059	*AC*-element
	AtMYB071 / MYB305	*AC*-element
	AtMYB072	*AC*-element
	AtMYB083	*AC*-element
	AtMYB088	FLP/MYB88 Binding site
	AtMYB091 / AS1	MYB-core type I and MYB-core Like
	AtMYB099	*AC*-element
	AtMYB103	*AC*-element
	AtMYB124 / FLP	FLP/MYB88 Binding site
