# Supplementary material for: Analysis of the DNA-Binding Activities of the Arabidopsis R2R3-MYB Transcription Factor Family by One-Hybrid Experiments in Yeast
Source: PLoS One. 2015 Oct 20;10(10):e0141044. doi: 10.1371/journal.pone.0141044 (PMC4613820; doi:10.1371/journal.pone.0141044)
Supplement: S2 Table — (PDF) [file pone.0141044.s008.pdf]

S2 Table: Details of previously described interactions between R2R3-MYBs and consensus DNA motifs in *Arabidopsis thaliana*.
(NB: Reference details are given in the main text or in S3 Table).

R2R3-MYB	*cis*-element						Reference
	type	Name	Sequence	Consensus	Assay	Promoter	
AtMYB000 / GL1	MYB-core type II	x	*AAAGTTAGTTA*	*TNGTTR*	EMSA	*x*	Romero *et al.*, 1998 - Plant J
AtMYB000 / GL1	MYB-core type II	MST m2	*aaaGTTAGTTgga* (R)	*TNGTTR*	Y1H, *in planta*	*AtTTG2*	Ishida *et al.*, 2007 - Plant Cell
AtMYB000 / GL1	MYB-core type II	MST m3	*acgTTAGTTgaa* (R)	*TNGTTR*	Y1H, *in planta*	*AtTTG2*	Ishida *et al.*, 2007 - Plant Cell
AtMYB002	MYB-core type I	x	*gcCAGTTAgt*	*CNGTTR*	EMSA	*x*	Urao *et al.*, 1996 - Plant J
AtMYB002	*AC*-element	GT-motif	*aaAAACCAaat* (R)	*ACC(A/T)A(A/C)(T/C)*	EMSA, Protoplasts	*AtADH1*	Hoeren *et al.*, 1998 - Genetics
AtMYB002	*AC*-element	SMRE3	*ACCAAACAT*	*ACC(A/T)A(A/C)(T/C)*	Y1H	x	Dubos *et al.*, 2014 - BMC Genom
AtMYB002	*AC*-element	AC-III / SMRE7	*CACCTAAC*	*ACC(A/T)A(A/C)(T/C)*	Y1H	x	Dubos *et al.*, 2014 - BMC Genom
AtMYB013	MYB-core type I	MBSI	*gtCAGTTAgggt*	*CNGTTR*	EMSA	*x*	Kirik *et al.*, 1998 - Plant J
AtMYB013	MYB-core type II	MBSII	*GTTAgtta*	*TNGTTR*	EMSA	*x*	Kirik *et al.*, 1998 - Plant J
AtMYB015	*AC*-element	x	*CACCTACCG* (R)	*ACC(A/T)A(A/C)(T/C)*	EMSA	*x*	Romero *et al.*, 1998 - Plant J
AtMYB015	*AC*-element	x	*CACCTATCG* (R)	*x*	EMSA	*x*	Romero *et al.*, 1998 - Plant J
AtMYB015	*AC*-element	x	*CATCTACCG* (R)	*x*	EMSA	*x*	Romero *et al.*, 1998 - Plant J
AtMYB019	*AC*-element	x	*ACCACAACC* (R)	*ACC(A/T)(A/C/T)(A/C/T)*	Y1H	x	Dubos *et al.*, 2014 - BMC Genom
AtMYB019	*AC*-element	AC-III / SMRE7	*CACCTAAC*	*ACC(A/T)A(A/C)(T/C)*	Y1H	x	Dubos *et al.*, 2014 - BMC Genom
AtMYB019	*AC*-element	AC-II / SMRE4	*CTAACCAACTAA* (R)	*ACC(A/T)(A/C/T)(A/C/T)*	Y1H	x	Dubos *et al.*, 2014 - BMC Genom
AtMYB021	MYB-core Like	*x*	*CAGTTC*	*x*	EMSA	x	S5 Fig.
AtMYB021	MYB-core Like	*x*	*CATTTA*	*x*	EMSA	x	S5 Fig.
AtMYB021	MYB-core Like	*x*	*CCATTC*	*x*	EMSA	x	S5 Fig.
AtMYB021	MYB-core Like	*x*	*CCGTTT*	*x*	EMSA	x	S5 Fig.
AtMYB021	MYB-core Like	*x*	*CAGTAA*	*x*	EMSA	x	S5 Fig.
AtMYB021	MYB-core Like	*x*	*CGATTA*	*x*	EMSA	x	S5 Fig.
AtMYB021	MYB-core Like	*x*	*CGGTAA*	*x*	EMSA	x	S5 Fig.
AtMYB021	MYB-core type I	*x*	*CCGTTA*	*CNGTTR*	EMSA	x	S5 Fig.
AtMYB021	MYB-core Like	*x*	*CGGTTC*	*x*	EMSA	x	S5 Fig.
AtMYB021	MYB-core type I	*x*	*CGGTTG*	*CNGTTR*	EMSA	x	S5 Fig.
AtMYB021	MYB-core Like	*x*	*CTCTTG*	*x*	EMSA	x	S5 Fig.
AtMYB021	MYB-core Like	*x*	*CTGTAC*	*x*	EMSA	x	S5 Fig.
AtMYB021	MYB-core Like	*x*	*TCGTCA*	*x*	EMSA	x	S5 Fig.
AtMYB021	MYB-core type II	*x*	*TCGTTA*	*TNGTTR*	EMSA	x	S5 Fig.
AtMYB021	MYB-core Like	*x*	*TGGTGG*	*x*	EMSA	x	S5 Fig.
AtMYB021	MYB-core Like	*x*	*TGGTTT*	*x*	EMSA	x	S5 Fig.
AtMYB021	MYB-core Like	*x*	*TGTTCT*	*x*	EMSA	x	S5 Fig.
AtMYB023	MYB-core type II	WBS I-L1	*ttaTcttGTTagttg*	*TNGTTR*	EMSA	*AtMYB23*	Kang *et al.*, 2009 - Plant Cell
AtMYB023	MYB-core type II	WBS II-L2	*ttagttaGTTGtggcaaa* (R)	*TNGTTR (x2)*	EMSA	*AtMYB23*	Kang *et al.*, 2009 - Plant Cell
AtMYB023	MYB-core type II	WBS I/II-L1	*tattaGTTGaaaac* (R)	*TNGTTR*	EMSA	*AtMYB23*	Kang *et al.*, 2009 - Plant Cell
AtMYB023	*AC*-element	WBS II-L1	*aaacCAACaaga*	*ACC(A/T)(A/C/T)(A/C/T)*	EMSA	*AtMYB23*	Kang *et al.*, 2009 - Plant Cell
AtMYB023	MYB-core type II	WBS II-L1	*tcttGTTGgttt* (R)	*TNGTTR*	EMSA	*AtMYB23*	Kang *et al.*, 2009 - Plant Cell
AtMYB023	MYB-core type II	WBS II-L1	*ggcttGTTGactct* (R)	*TNGTTR*	EMSA	*AtMYB23*	Kang *et al.*, 2009 - Plant Cell
AtMYB030	MYB-core type II	MYB1	*GTTTGTTa*	*TNGTTR*	EMSA	*AtSAUR-AC1*	Li *et al.*, 2009 - Plant J
AtMYB031 / ATY13	*AC*-element	x	*ACCACAACC* (R)	*ACC(A/T)(A/C/T)(A/C/T)*	Y1H, Protoplasts	x	Dubos *et al.*, 2014 - BMC Genom
AtMYB031 / ATY13	*AC*-element	AC-III / SMRE7	*CACCTAAC*	*ACC(A/T)A(A/C)(T/C)*	Y1H, Protoplasts	x	Dubos *et al.*, 2014 - BMC Genom
AtMYB031 / ATY13	*AC*-element	AC-II / SMRE4	*CTAACCAACTAA* (R)	*ACC(A/T)(A/C/T)(A/C/T)*	Y1H	x	Dubos *et al.*, 2014 - BMC Genom
AtMYB033	*AC*-element	AC-II / SMRE4	*CTAACCAACTAA* (R)	*ACC(A/T)(A/C/T)(A/C/T)*	Y1H	x	Dubos *et al.*, 2014 - BMC Genom
AtMYB044 / AtMYBR1	MYB-core type I	MBSI	*gtCAGTTAggg*	*CNGTTR*	EMSA	x	Kirik *et al.*, 1998 - Plant Mol Biol
AtMYB044 / AtMYBR1	MYB-core type II	MBSII	*aagtTAGTTAcg*	*TNGTTR*	EMSA	x	Kirik *et al.*, 1998 - Plant Mol Biol
AtMYB044 / AtMYBR1	MYB-core type I	x	*tcCGGTTAta*	*CNGTTR*	EMSA, *in planta*	*AtWRKY70*	Shim *et al.*, 2013 - Plant J
AtMYB044 / AtMYBR1	MYB-core type I	x	*CGGTTA*	*CNGTTR*	Oligo Arrays	*AtERA1*	Jung *et al.*, 2012 - Mol Cells
AtMYB044 / AtMYBR1	MYB-core type I	x	*CAGTTA*	*CNGTTR*	Oligo Arrays, EMSA	*AtABI5*, *AtERD1*, *AtFCA*	Jung *et al.*, 2012 - Mol Cells
AtMYB044 / AtMYBR1	MYB-core type I	x	*CCGTTA*	*CNGTTR*	Oligo Arrays	*x*	Jung *et al.*, 2012 - Mol Cells
AtMYB044 / AtMYBR1	MYB-core type I	x	*CCGTTT*	*CNGTTR*	Oligo Arrays	*x*	Jung *et al.*, 2012 - Mol Cells
AtMYB044 / AtMYBR1	MYB-core type I	x	*CCGTTG*	*CNGTTR*	Oligo Arrays	*x*	Jung *et al.*, 2012 - Mol Cells

AtMYB044 / AtMYBR1	MYB-core Like	x	CAGTTC	x	Oligo Arrays	x	Jung *et al.*, 2012 - Mol Cells
AtMYB044 / AtMYBR1	MYB-core Like	x	CCGGTA	x	Oligo Arrays	x	Jung *et al.*, 2012 - Mol Cells
AtMYB044 / AtMYBR1	MYB-core Like	x	CGGTAT	x	Oligo Arrays	x	Jung *et al.*, 2012 - Mol Cells
AtMYB044 / AtMYBR1	MYB-core type II	x	TAGTTG	TNGTTR	Oligo Arrays	x	Jung *et al.*, 2012 - Mol Cells
AtMYB044 / AtMYBR1	MYB-core Like	x	CAGGTA	x	Oligo Arrays	x	Jung *et al.*, 2012 - Mol Cells
AtMYB044 / AtMYBR1	MYB-core Like	x	AGGTAA	x	Oligo Arrays	x	Jung *et al.*, 2012 - Mol Cells
AtMYB044 / AtMYBR1	MYB-core Like	x	CGGTAA	x	Oligo Arrays	x	Jung *et al.*, 2012 - Mol Cells
AtMYB044 / AtMYBR1	MYB-core Like	x	CTGGTA	x	Oligo Arrays	x	Jung *et al.*, 2012 - Mol Cells
AtMYB044 / AtMYBR1	MYB-core Like	x	CGGGTA	x	Oligo Arrays	x	Jung *et al.*, 2012 - Mol Cells
AtMYB044 / AtMYBR1	MYB-core type I	x	CCGTTG	CNGTTR	Oligo Arrays	x	Jung *et al.*, 2012 - Mol Cells
AtMYB045	*AC*-element	x	ACCACAACC (R)	ACC(A/T)(A/C/T)(A/C/T)	Y1H	x	Dubos *et al.*, 2014 - BMC Genom
AtMYB045	*AC*-element	AC-II / SMRE4	CTAACCAACTAA (R)	ACC(A/T)(A/C/T)(A/C/T)	Y1H	x	Dubos *et al.*, 2014 - BMC Genom
AtMYB046	*AC*-element	M46RE	CACCTACT	(C/T)ACC(A/T)A(A/C)(C/T)	EMSA, Protoplasts	AtC3H14	Kim *et al.*, 2012 - Plant Mol Biol
AtMYB046	*AC*-element	SMRE1	ACCAAAT	ACC(A/T)A(A/C)(T/C)	EMSA, Protoplasts	AtMYB063	Zhong and Ye, 2012 - Plant Cell Physiol
AtMYB046	*AC*-element	SMRE2	ACCAACT	ACC(A/T)A(A/C)(T/C)	EMSA, Protoplasts	x	Zhong and Ye, 2012 - Plant Cell Physiol
AtMYB046	*AC*-element	SMRE3	ACCAAAC	ACC(A/T)A(A/C)(T/C)	EMSA, Protoplasts	x	Zhong and Ye, 2012 - Plant Cell Physiol
AtMYB046	*AC*-element	AC-II / SMRE4	ACCAACC	ACC(A/T)A(A/C)(T/C)	EMSA, Protoplasts	x	Zhong and Ye, 2012 - Plant Cell Physiol
AtMYB046	*AC*-element	SMRE5	ACCTAAT	ACC(A/T)A(A/C)(T/C)	EMSA, Protoplasts	x	Zhong and Ye, 2012 - Plant Cell Physiol
AtMYB046	*AC*-element	SMRE6	ACCTACT	ACC(A/T)A(A/C)(T/C)	EMSA, Protoplasts	x	Zhong and Ye, 2012 - Plant Cell Physiol
AtMYB046	*AC*-element	AC-III / SMRE7	ACCTAAC	ACC(A/T)A(A/C)(T/C)	EMSA, Protoplasts	x	Zhong and Ye, 2012 - Plant Cell Physiol
AtMYB046	*AC*-element	AC-I / SMRE8	ACCTACC	ACC(A/T)A(A/C)(T/C)	EMSA, Protoplasts	x	Zhong and Ye, 2012 - Plant Cell Physiol
AtMYB046	*AC*-element	AC-III / SMRE7	TACCTAAC (R)	ACC(A/T)A(A/C)(T/C)	Oligo Arrays	x	Franco-Zorilla *et al.*, 2014 - PNAS
AtMYB046	*AC*-element	x	AACCACC (R)	ACC(A/T)(A/C/T)(A/C/T)	Oligo Arrays	x	Franco-Zorilla *et al.*, 2014 - PNAS
AtMYB049	*AC*-element	x	ACCACAACC (R)	ACC(A/T)(A/C/T)(A/C/T)	Y1H	x	Dubos *et al.*, 2014 - BMC Genom
AtMYB049	*AC*-element	AC-III / SMRE7	CACCTAAC	ACC(A/T)A(A/C)(T/C)	Y1H	x	Dubos *et al.*, 2014 - BMC Genom
AtMYB049	*AC*-element	AC-II / SMRE4	CTAACCAACTAA (R)	ACC(A/T)(A/C/T)(A/C/T)	Y1H	x	Dubos *et al.*, 2014 - BMC Genom
AtMYB050	*AC*-element	x	ACCACAACC (R)	ACC(A/T)(A/C/T)(A/C/T)	Y1H	x	Dubos *et al.*, 2014 - BMC Genom
AtMYB050	*AC*-element	AC-III / SMRE7	CACCTAAC	ACC(A/T)A(A/C)(T/C)	Y1H	x	Dubos *et al.*, 2014 - BMC Genom
AtMYB050	*AC*-element	AC-II / SMRE4	CTAACCAACTAA (R)	ACC(A/T)(A/C/T)(A/C/T)	Y1H	x	Dubos *et al.*, 2014 - BMC Genom
AtMYB052	MYB-core type I	x	CCGTTA	CNGTTR	Oligo Arrays	x	Franco-Zorilla *et al.*, 2014 - PNAS
AtMYB052	MYB-core type II	x	GTTAGTTG	TNGTTR	Oligo Arrays	x	Franco-Zorilla *et al.*, 2014 - PNAS
AtMYB055	*AC*-element	SMRE8 / AC-I	ACCTACCG (R)	ACC(A/T)A(A/C)(T/C)	Oligo Arrays	x	Franco-Zorilla *et al.*, 2014 - PNAS
AtMYB055	*AC*-element	x	CAACCACC (R)	ACC(A/T)(A/C/T)(A/C/T)	Oligo Arrays	x	Franco-Zorilla *et al.*, 2014 - PNAS
AtMYB058	*AC*-element	x	ACCACAACC (R)	ACC(A/T)(A/C/T)(A/C/T)	Y1H	x	Dubos *et al.*, 2014 - BMC Genom
AtMYB058	*AC*-element	AC-III / SMRE7	CACCTAAC	ACC(A/T)A(A/C)(T/C)	Y1H	x	Dubos *et al.*, 2014 - BMC Genom
AtMYB058	*AC*-element	AC-II / SMRE4	CTAACCAACTAA (R)	ACC(A/T)(A/C/T)(A/C/T)	Y1H	x	Dubos *et al.*, 2014 - BMC Genom
AtMYB059	*AC*-element	AC-III / SMRE7	ACCTAAC (R)	ACC(A/T)A(A/C)(T/C)	Oligo Arrays	x	Franco-Zorilla *et al.*, 2014 - PNAS
AtMYB060	*AC*-element	x	ACCACAACC (R)	ACC(A/T)(A/C/T)(A/C/T)	Y1H	x	Dubos *et al.*, 2014 - BMC Genom
AtMYB060	*AC*-element	AC-III / SMRE7	CACCTAAC	ACC(A/T)A(A/C)(T/C)	Y1H	x	Dubos *et al.*, 2014 - BMC Genom
AtMYB060	*AC*-element	AC-II / SMRE4	CTAACCAACTAA (R)	ACC(A/T)(A/C/T)(A/C/T)	Y1H	x	Dubos *et al.*, 2014 - BMC Genom
AtMYB061	*AC*-element	AC-III / SMRE7	cACCTAAC	ACC(A/T)A(A/C)(T/C)	EMSA, Y1H	AtKNAT7	Romano *et al.*, 2012 - New Phytol
AtMYB061	*AC*-element	AC-I / SMRE8	ACCTAC	ACC(A/T)(A/C/T)(A/C/T)	EMSA, Y1H	x	Prouse and Campbell, 2012 - PLOS one
AtMYB061	*AC*-element	x	ACCAAT	ACC(A/T)(A/C/T)(A/C/T)	EMSA, Y1H	x	Prouse and Campbell, 2012 - PLOS one
AtMYB061	*AC*-element	x	ACCAAA	ACC(A/T)(A/C/T)(A/C/T)	EMSA, Y1H	x	Prouse and Campbell, 2012 - PLOS one
AtMYB061	*AC*-element	x	ACCATA	ACC(A/T)(A/C/T)(A/C/T)	EMSA, Y1H	x	Prouse and Campbell, 2012 - PLOS one
AtMYB061	*AC*-element	AC-II / SMRE4	ACCAAC	ACC(A/T)(A/C/T)(A/C/T)	EMSA, Y1H	x	Prouse and Campbell, 2012 - PLOS one
AtMYB061	*AC*-element	x	ACCACA	ACC(A/T)(A/C/T)(A/C/T)	EMSA, Y1H	x	Prouse and Campbell, 2012 - PLOS one
AtMYB061	*AC*-element	x	ACCACC	ACC(A/T)(A/C/T)(A/C/T)	EMSA, Y1H	x	Prouse and Campbell, 2012 - PLOS one
AtMYB061	*AC*-element	x	ACCACAACC (R)	ACC(A/T)(A/C/T)(A/C/T)	Y1H, Protoplasts	x	Dubos *et al.*, 2014 - BMC Genom
AtMYB061	*AC*-element	AC-III / SMRE7	CACCTAAC	ACC(A/T)A(A/C)(T/C)	Y1H, Protoplasts	x	Dubos *et al.*, 2014 - BMC Genom
AtMYB061	*AC*-element	AC-II / SMRE4	CTAACCAACTAA (R)	ACC(A/T)(A/C/T)(A/C/T)	Y1H	x	Dubos *et al.*, 2014 - BMC Genom
AtMYB066 / WER	MYB-core type II	WBS I-L1	ttaTcttGTTagttg	TNGTTR	EMSA, *in planta*	AtMYB23	Kang *et al.*, 2009 - Plant Cell
AtMYB066 / WER	MYB-core type II	WBS II-L2	ttagttaGTTGtggcaaa (R)	TNGTTR (x2)	EMSA	AtMYB23	Kang *et al.*, 2009 - Plant Cell
AtMYB066 / WER	MYB-core type II	WBS I/II-L1	tattaGTTGaaaac (R)	TNGTTR	EMSA	AtMYB23	Kang *et al.*, 2009 - Plant Cell
AtMYB066 / WER	*AC*-element	WBS II-L1	aaacCAACaaga	ACC(A/T)(A/C/T)(A/C/T)	EMSA	AtMYB23	Kang *et al.*, 2009 - Plant Cell
AtMYB066 / WER	MYB-core type II	WBS II-L1	tcttGTTGgttt (R)	TNGTTR	EMSA	AtMYB23	Kang *et al.*, 2009 - Plant Cell

AtMYB066 / WER	MYB-core type II	WBS II-L1	*ggcttGTTGactct* (R)	*TNGTTR*	EMSA	*AtMYB23*	Kang *et al.*, 2009 - Plant Cell
AtMYB066 / WER	MYB-core type II	MST m2	*aaaGTTAGTTgga* (R)	*TNGTTR*	Y1H, *in planta*	*AtTTG2*	Ishida *et al.*, 2007 - Plant Cell
AtMYB066 / WER	MYB-core type II	MST m3	*acgTTAGTTgaa* (R)	*TNGTTR*	Y1H, *in planta*	*AtTTG2*	Ishida *et al.*, 2007 - Plant Cell
AtMYB066 / WER	MYB-core type I	CPC MBS1	*gacAGTTGGAGaaa* (R)	*CNGTTR*	EMSA	*AtCPC*	Koshino-Kimura *et al.*, 2005 - Plant Cell Physiol
AtMYB066 / WER	MYB-core type I	GL2 MBS1	*cttaCCGTTAgtc* (R)	*CNGTTR*	EMSA, *in planta*	*AtGL2*	Koshino-Kimura *et al.*, 2005 - Plant Cell Physiol
AtMYB066 / WER	MYB-core type I	GL2 MBS2	*tataCTGTTAgta* (R)	*CNGTTR*	EMSA, *in planta*	*AtGL2*	Koshino-Kimura *et al.*, 2005 - Plant Cell Physiol
AtMYB066 / WER	MYB-core type I	GWBSI	aaaTgcgGTTgg	*CNGTTR*	EMSA, Y1H	*AtGL2*	Song *et al.*, 2011- Plant Physiol
AtMYB066 / WER	MYB-core type II	GWBSII	aaGTTaGTTga	*TNGTTR*	EMSA, Y1H	*AtGL2*	Song *et al.*, 2011- Plant Physiol
AtMYB071 / MYB305	*AC*-element	SMRE3	*ACCAAACAT*	*ACC(A/T)A(A/C)(T/C)*	Y1H	x	Dubos *et al.*, 2014 - BMC Genom
AtMYB072	*AC*-element	AC-III / SMRE7	*CACCTAAC*	*ACC(A/T)A(A/C)(T/C)*	Y1H	x	Dubos *et al.*, 2014 - BMC Genom
AtMYB073	*AC*-element	AC-II / SMRE4	*CTAACCAACTAA* (R)	*ACC(A/T)(A/C/T)(A/C/T)*	Y1H	x	Dubos *et al.*, 2014 - BMC Genom
AtMYB075 / PAP1	*AC*-element	x	*ACCACAACC* (R)	*ACC(A/T)(A/C/T)(A/C/T)*	Y1H	x	Dubos *et al.*, 2014 - BMC Genom
AtMYB077 / AtMYBR2	MYB-core type II	x	*AAAGTTAGTTA*	*TNGTTR*	EMSA	*x*	Romero *et al.*, 1998 - Plant J
AtMYB077 / AtMYBR2	MYB-core type I	x	*AAAAAACGGTTA*	*CNGTTR*	EMSA	*x*	Romero *et al.*, 1998 - Plant J
AtMYB077 / AtMYBR2	MYB-core type I	x	*TGACAGTTA*	*CNGTTR*	EMSA	*x*	Romero *et al.*, 1998 - Plant J
AtMYB077 / AtMYBR2	MYB-core type I	x	*CAGTTA* (R)	*CNGTTR*	Oligo Arrays	*x*	Oh *et al.*, 2012 - J Korean Soc Appl Biol Chem
AtMYB077 / AtMYBR2	MYB-core type I	x	*CAGTTG* (R)	*CNGTTR*	Oligo Arrays	*x*	Oh *et al.*, 2012 - J Korean Soc Appl Biol Chem
AtMYB077 / AtMYBR2	MYB-core type I	x	*CCGTTA* (R)	*CNGTTR*	Oligo Arrays	*x*	Oh *et al.*, 2012 - J Korean Soc Appl Biol Chem
AtMYB077 / AtMYBR2	MYB-core Like	x	*CCGGTA* (R)	*x*	Oligo Arrays	*x*	Oh *et al.*, 2012 - J Korean Soc Appl Biol Chem
AtMYB077 / AtMYBR2	MYB-core type I	x	*CCGTTG* (R)	*CNGTTR*	Oligo Arrays	*x*	Oh *et al.*, 2012 - J Korean Soc Appl Biol Chem
AtMYB077 / AtMYBR2	MYB-core Like	x	*CCGTTT* (R)	x	Oligo Arrays	*x*	Oh *et al.*, 2012 - J Korean Soc Appl Biol Chem
AtMYB077 / AtMYBR2	MYB-core type I	x	*CGGTTA* (R)	*CNGTTR*	Oligo Arrays	*x*	Oh *et al.*, 2012 - J Korean Soc Appl Biol Chem
AtMYB077 / AtMYBR2	MYB-core type I	x	*CGGTTG* (R)	*CNGTTR*	Oligo Arrays	*x*	Oh *et al.*, 2012 - J Korean Soc Appl Biol Chem
AtMYB077 / AtMYBR2	MYB-core Like	x	*CGGTTT* (R)	x	Oligo Arrays	*x*	Oh *et al.*, 2012 - J Korean Soc Appl Biol Chem
AtMYB077 / AtMYBR2	MYB-core type I	x	*CTGTTA* (R)	*CNGTTR*	Oligo Arrays	*x*	Oh *et al.*, 2012 - J Korean Soc Appl Biol Chem
AtMYB077 / AtMYBR2	MYB-core type II	x	*TAGTTA* (R)	*TNGTTR*	Oligo Arrays	*x*	Oh *et al.*, 2012 - J Korean Soc Appl Biol Chem
AtMYB077 / AtMYBR2	MYB-core Like	x	*TAGTTT* (R)	x	Oligo Arrays	*x*	Oh *et al.*, 2012 - J Korean Soc Appl Biol Chem
AtMYB077 / AtMYBR2	MYB-core type II	x	*TCGTTA* (R)	*TNGTTR*	Oligo Arrays	*x*	Oh *et al.*, 2012 - J Korean Soc Appl Biol Chem
AtMYB081	*AC*-element	AC-II / SMRE4	*CTAACCAACTAA* (R)	*ACC(A/T)(A/C/T)(A/C/T)*	Y1H	x	Dubos *et al.*, 2014 - BMC Genom
AtMYB083	*AC*-element	SMRE1	*ACCAAAT*	*ACC(A/T)A(A/C)(T/C)*	EMSA, Protoplasts	*AtMYB063*	Zhong and Ye, 2012 - Plant Cell Physiol
AtMYB083	*AC*-element	SMRE2	*ACCAACT*	*ACC(A/T)A(A/C)(T/C)*	EMSA, Protoplasts	*x*	Zhong and Ye, 2012 - Plant Cell Physiol
AtMYB083	*AC*-element	SMRE3	*ACCAAAC*	*ACC(A/T)A(A/C)(T/C)*	EMSA, Protoplasts	*x*	Zhong and Ye, 2012 - Plant Cell Physiol
AtMYB083	*AC*-element	AC-II / SMRE4	*ACCAACC*	*ACC(A/T)A(A/C)(T/C)*	EMSA, Protoplasts	*x*	Zhong and Ye, 2012 - Plant Cell Physiol
AtMYB083	*AC*-element	SMRE5	*ACCTAAT*	*ACC(A/T)A(A/C)(T/C)*	EMSA, Protoplasts	*x*	Zhong and Ye, 2012 - Plant Cell Physiol
AtMYB083	*AC*-element	SMRE6	*ACCTACT*	*ACC(A/T)A(A/C)(T/C)*	EMSA, Protoplasts	*x*	Zhong and Ye, 2012 - Plant Cell Physiol
AtMYB083	*AC*-element	AC-III / SMRE7	*ACCTAAC*	*ACC(A/T)A(A/C)(T/C)*	EMSA, Protoplasts	*x*	Zhong and Ye, 2012 - Plant Cell Physiol
AtMYB083	*AC*-element	AC-I / SMRE8	*ACCTACC*	*ACC(A/T)A(A/C)(T/C)*	EMSA, Protoplasts	*x*	Zhong and Ye, 2012 - Plant Cell Physiol
AtMYB084	*AC*-element	x	*CACCTACCG* (R)	*ACC(A/T)A(A/C)(T/C)*	EMSA	*x*	Romero *et al.*, 1998 - Plant J
AtMYB084	*AC*-element	x	*CACCTATCG* (R)	*x*	EMSA	*x*	Romero *et al.*, 1998 - Plant J
AtMYB086	*AC*-element	x	*ACCACAACC* (R)	*ACC(A/T)(A/C/T)(A/C/T)*	Y1H	x	Dubos *et al.*, 2014 - BMC Genom
AtMYB086	*AC*-element	AC-III / SMRE7	*CACCTAAC*	*ACC(A/T)A(A/C)(T/C)*	Y1H	x	Dubos *et al.*, 2014 - BMC Genom
AtMYB086	*AC*-element	AC-II / SMRE4	*CTAACCAACTAA* (R)	*ACC(A/T)(A/C/T)(A/C/T)*	Y1H	x	Dubos *et al.*, 2014 - BMC Genom
AtMYB088	FLP/MYB88 Binding site	x	*gaaGCGGGaaa*	*(A/T/G)(A/T/G)C(C/G)(C/G)*	EMSA	*AtCDKB1;1*	Xie et al., 2010 - Plant Cell
AtMYB091 / AS1	MYB-core type I	x	*CTGTTA*	*CNGTTR*	EMSA, ChIP	*AtBP*	Guo et al., 2008 - Plant Cell
AtMYB091 / AS1	MYB-core Like	x	*CTGTTT*	*x*	EMSA, ChIP	*AtBP*	Guo et al., 2008 - Plant Cell
AtMYB091 / AS1	MYB-core type I	x	*caCAGTTGga*	*CNGTTR*	ChIP	*AtKNAT2*	Guo et al., 2008 - Plant Cell
AtMYB091 / AS1	MYB-core type I	x	*ttCTGTTAct*	*CNGTTR*	ChIP	*AtKNAT2*	Guo et al., 2008 - Plant Cell
AtMYB092	*AC*-element	x	*ACCACAACC* (R)	*ACC(A/T)(A/C/T)(A/C/T)*	Y1H	x	Dubos *et al.*, 2014 - BMC Genom
AtMYB092	*AC*-element	AC-III / SMRE7	*CACCTAAC*	*ACC(A/T)A(A/C)(T/C)*	Y1H	x	Dubos *et al.*, 2014 - BMC Genom
AtMYB092	*AC*-element	AC-II / SMRE4	*CTAACCAACTAA* (R)	*ACC(A/T)(A/C/T)(A/C/T)*	Y1H	x	Dubos *et al.*, 2014 - BMC Genom
AtMYB094	*AC*-element	x	*ACCACAACC* (R)	*ACC(A/T)(A/C/T)(A/C/T)*	Y1H	x	Dubos *et al.*, 2014 - BMC Genom
AtMYB094	*AC*-element	AC-III / SMRE7	*CACCTAAC*	*ACC(A/T)A(A/C)(T/C)*	Y1H	x	Dubos *et al.*, 2014 - BMC Genom
AtMYB094	*AC*-element	AC-II	*CTAACCAACTAA* (R)	*ACC(A/T)(A/C/T)(A/C/T)*	Y1H	x	Dubos *et al.*, 2014 - BMC Genom
AtMYB096	MYB-core type II	x	*tcTAGTTAtt* (R)	*TNGTTR*	EMSA, Protoplasts	*AtKCS1*	Seo *et al.*, 2011 - Plant Cell
AtMYB096	MYB-core type II	x	*tcTAGTTAgt* (R)	*TNGTTR*	EMSA, Protoplasts	*AtKCS1*	Seo *et al.*, 2011 - Plant Cell
AtMYB096	MYB-core type II	x	*agTAGTTAgg* (R)	*TNGTTR*	EMSA, Protoplasts	*AtKCS2*	Seo *et al.*, 2011 - Plant Cell

AtMYB096	MYB-core type II	x	*taTAGTTAgt* (R)	*TNGTTR*	EMSA, Protoplasts	*AtKCS2*	Seo *et al.*, 2011 - Plant Cell
AtMYB096	MYB-core type I	x	*atCAGTTAat* (R)	*CNGTTR*	EMSA, Protoplasts	*AtKCS6*	Seo *et al.*, 2011 - Plant Cell
AtMYB096	MYB-core type I	x	*tgCAGTTAag*	*CNGTTR*	EMSA, Protoplasts	*AtKCR1*	Seo *et al.*, 2011 - Plant Cell
AtMYB096	MYB-core type I	x	*tgCAGTTAtc* (R)	*CNGTTR*	EMSA, Protoplasts	*AtKCR1*	Seo *et al.*, 2011 - Plant Cell
AtMYB099	*AC*-element	AC-III / SMRE7	*CACCTAAC*	*ACC(A/T)A(A/C)(T/C)*	Y1H	x	Dubos *et al.*, 2014 - BMC Genom
AtMYB102 / AtM4	*AC*-element	AC-II	*CTAACCAACTAA* (R)	*ACC(A/T)(A/C/T)(A/C/T)*	Y1H	x	Dubos *et al.*, 2014 - BMC Genom
AtMYB103	*AC*-element	x	*ACCACAACC* (R)	*ACC(A/T)(A/C/T)(A/C/T)*	Y1H	x	Dubos *et al.*, 2014 - BMC Genom
AtMYB103	*AC*-element	AC-III / SMRE7	*CACCTAAC*	*ACC(A/T)A(A/C)(T/C)*	Y1H	x	Dubos *et al.*, 2014 - BMC Genom
AtMYB103	*AC*-element	AC-II / SMRE4	*CTAACCAACTAA* (R)	*ACC(A/T)(A/C/T)(A/C/T)*	Y1H	x	Dubos *et al.*, 2014 - BMC Genom
AtMYB107	*AC*-element	x	*ACCACAACC* (R)	*ACC(A/T)(A/C/T)(A/C/T)*	Y1H	x	Dubos *et al.*, 2014 - BMC Genom
AtMYB107	*AC*-element	AC-III / SMRE7	*CACCTAAC*	*ACC(A/T)A(A/C)(T/C)*	Y1H	x	Dubos *et al.*, 2014 - BMC Genom
AtMYB108 / BOS1	*AC*-element	SMRE3	*ACCAAACAT*	*ACC(A/T)A(A/C)(T/C)*	Y1H	x	Dubos *et al.*, 2014 - BMC Genom
AtMYB108 / BOS1	*AC*-element	AC-III / SMRE7	*CACCTAAC*	*ACC(A/T)A(A/C)(T/C)*	Y1H	x	Dubos *et al.*, 2014 - BMC Genom
AtMYB111 / PFG3	*AC*-element	AC-I / SMRE8	*ACCTACC* (R)	*ACC(A/T)A(A/C)(T/C)*	Oligo Arrays	*x*	Franco-Zorilla *et al.*, 2014 - PNAS
AtMYB111 / PFG3	*AC*-element	AC-I / SMRE8	*CACCTACC* (R)	*ACC(A/T)A(A/C)(T/C)*	Oligo Arrays	*x*	Franco-Zorilla *et al.*, 2014 - PNAS
AtMYB111 / PFG3	*AC*-element	AC-II / SMRE4	*CTAACCAACTAA* (R)	*ACC(A/T)(A/C/T)(A/C/T)*	Y1H	x	Dubos *et al.*, 2014 - BMC Genom
AtMYB112	*AC*-element	SMRE3	*ACCAAACAT*	*ACC(A/T)A(A/C)(T/C)*	Y1H	x	Dubos *et al.*, 2014 - BMC Genom
AtMYB118	MYB-core type I	x	*CCGTTA* (R)	*CNGTTR*	EMSA, Protoplasts, *in planta*	*AtODD*	Barthole *et al.*, 2014 - Plant Cell
AtMYB123 / TT2	MYB-core type I	x	*CTGTTG*	*CNGTTR*	Y1H, Protoplasts, *in planta*	*AtBAN*	Thévenin *et al.*, 2012 - New Phytol
AtMYB123 / TT2	MYB-core type II	MST m2	*aaaGTTAGTTgga* (R)	*TNGTTR*	Y1H, *in planta*	*AtTTG2*	Ishida *et al.*, 2007 - Plant Cell
AtMYB123 / TT2	MYB-core type II	MST m3	*acgTTAGTTgaa* (R)	*TNGTTR*	Y1H, *in planta*	*AtTTG2*	Ishida *et al.*, 2007 - Plant Cell
AtMYB124 / FLP	FLP/MYB88 Binding site	SS-30	*GGCGCGCC*	*(A/T/G)(A/T/G)C(C/G)(C/G)*	EMSA	x	Xie *et al.*, 2010 - Plant Cell
AtMYB124 / FLP	FLP/MYB88 Binding site	x	*gaaGCGGGaaa*	*(A/T/G)(A/T/G)C(C/G)(C/G)*	EMSA, *in planta*	*AtCDKB1;1*	Xie *et al.*, 2010 - Plant Cell
