# Supplementary material for: Analysis of the DNA-Binding Activities of the Arabidopsis R2R3-MYB Transcription Factor Family by One-Hybrid Experiments in Yeast
Source: PLoS One. 2015 Oct 20;10(10):e0141044. doi: 10.1371/journal.pone.0141044 (PMC4613820; doi:10.1371/journal.pone.0141044)
Supplement: S3 Table — (PDF) [file pone.0141044.s009.pdf]

Table S3: DNA motifs analysed in this study

DNA motifs from which *trans*-activation has been observed in yeast one-hybrid experiments

Sequence	Type	reference
GGTTGGTG	*AC*-element	Hatton *et al.*, 1995 - Plant J
GGTAGGTG	*AC*-element	Hatton *et al.*, 1995 - Plant J
TAGTTA	MYB-core Type II	Romero *et al.*, 1998 - Plant J
AGGTTA	MYB-core like	Solano *et al.*, 1995 - EMBO J
ACAAGTTA	MYB-core Like	x
AGTGGTTA	MYB-core Type II	Solano *et al.*, 1995 - EMBO J
GACAGTTG	MYB-core Type I	Koshino-kimura *et al.*, 2005
TACTGTTG	MYB-core Type I	x
TGCGGTTG	MYB-core Type I	Song *et al.*, 2011- Plant Physiol
AGTAGTTA	MYB-core Type II	Solano *et al.*, 1995 - EMBO J
ATTAGTTG	MYB-core Type II	Kang *et al.*, 2009 - Plant Cell
AAAAGTTA	MYB-core Like	Solano *et al.*, 1995 - EMBO J
GTCAGTTA	MYB-core Type I	Solano *et al.*, 1995 - EMBO J
GTTAGTTG	MYB-core Type II	Song *et al.*, 2011- Plant Physiol
GCTTGTTG	MYB-core Type II	Kang *et al.*, 2009 - Plant Cell
GTTAGTTA	MYB-core Type II	Solano *et al.*, 1995 - EMBO J

References

- **D. Hatton, R. Sablowski, M.H. Yung, C. Smith, W. Schuch and M. Bevan** (1995) Two classes of cis sequences contribute to tissue-specific expression of a PAL2 promoter in transgenic tobacco. *Plant J*, **7**, 859-876.
- **Y.H. Kang, V. Kirik, M. Hulskamp, K.H. Nam, K. Hagely, M.M. Lee and J. Schiefelbein** (2009) The MYB23 gene provides a positive feedback loop for cell fate specification in the Arabidopsis root epidermis. *Plant Cell*, **21**, 1080-1094.
- **Y. Koshino-Kimura, T. Wada, T. Tachibana, R. Tsugeki, S. Ishiguro and K. Okada** (2005) Regulation of CAPRICE transcription by MYB proteins for root epidermis differentiation in Arabidopsis. *Plant Cell Physiol*, **46**, 817-826.
- **I. Romero, A. Fuertes, M.J. Benito, J.M. Malpica, A. Leyva and J. Paz-Ares** (1998) More than 80R2R3-MYB regulatory genes in the genome of Arabidopsis thaliana. *Plant J*, **14**, 273-284.
- **R. Solano, C. Nieto, J. Avila, L. Canas, I. Diaz and J. Paz-Ares** (1995) Dual DNA binding specificity of a petal epidermis-specific MYB transcription factor (MYB.Ph3) from Petunia hybrida. *Embo J*, **14**, 1773-1784.
- **S.K. Song, K.H. Ryu, Y.H. Kang, J.H. Song, Y.H. Cho, S.D. Yoo, J. Schiefelbein and M.M. Lee** (2011) Cell fate in the Arabidopsis root epidermis is determined by competition between WEREWOLF and CAPRICE. Plant Physiol, 157, 1196-1208.
