# Supplementary material for: Analysis of the DNA-Binding Activities of the Arabidopsis R2R3-MYB Transcription Factor Family by One-Hybrid Experiments in Yeast
Source: PLoS One. 2015 Oct 20;10(10):e0141044. doi: 10.1371/journal.pone.0141044 (PMC4613820; doi:10.1371/journal.pone.0141044)
Supplement: S4 Table — (PDF) [file pone.0141044.s010.pdf]

Table S4. Summary of yeast one-hybrid screens results. Each cell corresponds to an interaction between a R2R3-MYB protein and a DNA motif. Empty cells indicate that no interaction has been observed, whereas plus signs indicate a positive interaction (+ and ++ corresponding to a weak and strong interaction, respectively). Cells highlighted in yellow correspond to positive interactions already described in the literature that were confirmed in this study with either the full DNA sequence (bright yellow) or a part of it containing a MYB binding domain (light yellow). Cells highlighted in blue correspond to positive interactions described in the literature that were not confirmed in this study with either the full DNA sequence (dark blue) or a part of it containing a MYB binding domain (light blue). Cells highlighted in red correspond to negative interactions already described in the literature that were confirmed in this study with either the full DNA sequence (bright red or a part of it containing a MYB binding domain (light red). Cells highlighted in green correspond to negative interactions described in the literature that were not confirmed in this study with either the full DNA sequence (dark green) or a part of it containing a MYB binding domain (light green).
Stars indicate the DNA sequences that form an AC-rich element in between two consecutive DNA motifs (*:group IIa, **: from IId).

R2R3-MYB subgroup	DBD subgroup	gene name	gene code	goup I						group Iia		group Iib		group Iic				group Iid		Number of interacting DNA motifs
				TACTGTTG	TGCGGTTG	AAAAGTTA	GTCAGTTA	ACAAGTTA	ATTAGTTG	GCTTGTTG (*)	GTTAGTTG (*)	GGATGGTG	GGTTGGTG	AGGTTA	AGTAGTTA	AGTGGTTA	TAGTTA	GTTAGTTA (**)	GACAGTTG (**)	
S1	S1	AtMYB030	AT3G28910		++	++	++				++	++	+					++	++	8
S1	S1	AtMYB031	AT1G74650	++	++	++	++	++		++	+ [bright yellow]	++	+ [light yellow]						++	10
S1	S1	AtMYB060	AT1G08810.1	++	++	++	++				++ [bright yellow]	++	+ [light yellow]					++	++	9
S1	S1	AtMYB094	AT3G47600	++	++	++	++			++	++ [bright yellow]	++	+ [light yellow]					++	++	10
S1	S1	AtMYB096	AT5G62470.2	++	++	++	++ [light yellow]		++	++	++	++	+		[dark blue]		[light blue]	++ [light yellow]	++	11
S2	S2	AtMYB013	AT1G06180	++	++	++	++ [bright yellow]	++	++	++	++	++	++		++			[dark blue]		11
S2	S2	AtMYB014	AT2G31180	++	++	++	++	++	++	++	++	++	+		++	++		++	+	14
S2	S2	AtMYB015	AT3G23250.1	++	++	++	++		++ [light yellow]	++	++	++ [bright yellow]	++					[bright red]	+ [light green]	10
S3	S3	AtMYB058	AT1G16490		+	++	++				++ [bright yellow]	++	++ [light yellow]							6
S3	S3	AtMYB063	AT1G79180		+	++	++	++	++	++	+	++	++					++	+	11
**SU**	S3	AtMYB010	AT3G12820	++		++		+	++	++	++	++	++	++	++	++				11
**SU**	S3	AtMYB072	AT1G56160	++	++	++	++	+		++	++	++	+		++	++				11
S4	S4	AtMYB003	AT1G22640	++	+	++	++	++		++	+								+	8
S4	S4	AtMYB004	AT4G38620		+	+	++	++			+								+	6
S4	S4	AtMYB007	AT2G16720	++	++	++	++			++										5
S4	S4	AtMYB032	AT4G34990	++	++	++	++	++	++	++	+					+				9
**SU**	S4	AtMYB006	AT4G09460	++		++	++	++		++	++					++				7
**SU**	S4	AtMYB008	AT1G35515	++		++	++	+		++	++	+		++	++	++				10
S5	S5	AtMYB123	AT5G35550	++ [light yellow]	++	++	++	++	++	++	+ [bright yellow]					+	++		+	11
**SU**	S5	AtMYB005	AT3G13540	++	++	++	++	++	++	++				++	++			++		10
S6	S6	AtMYB075	AT1G56650	++	++	++	++	++	++	++		+							+	9
S6	S6	AtMYB090	AT1G66390	++	++	++	++	++	++	++						++				8
S6	S6	AtMYB113	AT1G66370	++	++	++	++	++	++	++						++	++	++		10
S6	S6	AtMYB114	AT1G66380	++	++	++	++	++	++	++						++	++			9
S7	S7	AtMYB011	AT3G62610	++	++	++	++	++	++	++						++	++			9
S7	S7	AtMYB111	AT5G49330	++	+	+	++		+		+ [bright yellow]	++ [bright yellow]	++ [light yellow]					++		9
S9	S9a	AtMYB016	AT5G15310.1	++	+	+	++	++	+			++	+							8
S9	S9b	AtMYB017	AT3G61250	++	+	+	++	++											+	6
S9	S9a	AtMYB106	AT3G01140	++	++	++	++	++		++										6
S10	S10	AtMYB009	AT5G16770	++	++	++	++	++	++	++		++	++	++	++		++			12
S10	S10	AtMYB039	AT4G17785	++	++	++	++	++	++	++		++					++			9
S10	S10	AtMYB107	AT3G02940	++	++	++	++	++	++	++						++		++		9
S11	S11	AtMYB041	AT4G28110	++	++	++	++	++	++	++				++	++	++		++		11
S11	S11	AtMYB074	AT4G05100	++	++	++	++	++	++	++		+		++		++				10
S11	S11	AtMYB102	AT4G21440	++	++	++	++	++	++	++	[dark blue]	+	[light blue]			++				9
**SU**	S11	AtMYB049	AT5G54230	++	+	++	++	++	++	++	++ [bright yellow]	++	+ [light yellow]	++	++	++	+	++		15
S12	S12	AtMYB028	AT5G61420.2	++	++	++	++	++	++			++	++		++	++				10
S12	S12	AtMYB029	AT5G07690	++	++	+	++	++	++			+	+	++	++					10
S12	S12	AtMYB034	AT5G60890	++	+	++	++		++						++	++				7
S12	S12	AtMYB051	AT1G18570	++	+	++	++	++	++			+			++		++		+	10
S12	S12	AtMYB076	AT5G07700	++	+	++	++					++	++		++				+	8
S12	S12	AtMYB122	AT1G74080	++	+	++	++	++		++				++						7
S13	S13	AtMYB050	AT1G57560	++	+	++	++	++	++	++	++ [bright yellow]	++	++ [light yellow]	++				++		12
S13	S13	AtMYB055	AT4G01680.1	++	++	++	++	++	++	++	+	[light blue]		++				++		10
S13	S13	AtMYB061	AT1G09540	++	++	++	++	++	++		++ [light yellow]	++ [bright yellow]	++ [bright yellow]					++		10
S13	S13	AtMYB086	AT5G26660	++	++	++	++	++	++	++	++ [bright yellow]	++	++ [light yellow]	++				++		12
S14	S14	AtMYB036	AT5G57620	++	++	++	++	++	++	++		+		++				++		10
S14	S14	AtMYB037	AT5G23000	++	++	+	++	++	++	++		+							+	9
S14	S14	AtMYB038	AT2G36890	++	++	+	++	++	++			+			++		++			9
S14	S14	AtMYB068	AT5G65790	++	++	++	++	++	++			++	+		++				+	10
S14	S14	AtMYB084	AT3G49690	+	++	+	++	++	[light red]			+ [bright yellow]	[light blue]				++	[bright red]	+ [light green]	8
S14	S14	AtMYB087	AT4G37780	++	++	+	++	++	++			+			++			++		9
S15	S15	AtMYB000	AT3G27920	++	+	++	++	++	++ [light green]		++ [bright yellow]	[light red]	[light red]		++			++ [bright yellow]	[light red]	9
S15	S15	AtMYB023	AT5G40330	++	++	++	++	++	[dark blue]	++ [bright yellow]	++ [bright yellow]		[dark blue]	++				++		9
S15	S15	AtMYB066	AT5G14750	++	++ [bright yellow]	++	++	++	[dark blue]	[dark blue]	++ [bright yellow]			++	++	++		++	[dark blue]	10
**SU**	S15	AtMYB082	AT5G52600	++	+	++	++	++	++	++	++		+	++	++	++	++	++		14
S16	S16	AtMYB018	AT4G25560	++	+	++	++	++	++		+	+		++	++	++				11
S16	S16	AtMYB019	AT5G52260	++	++	++	++	++	+		++	++	++ [bright yellow]			++		++		11
S16	S16	AtMYB045	AT3G48920	++	+	++	++	++	+	++	++	++	[light blue]		++			++		11
S18	S18	AtMYB033	AT5G06100.2	++	++	++	++	++	++	++	[dark blue]		[light blue]							7
S18	S18	AtMYB065	AT3G11440	+	++		++				++							++		5
S18	S18	AtMYB081	AT2G26960	++	++	++	++	++	++	++	++ [bright yellow]		[light blue]	++						9
S18	S18	AtMYB097	AT4G26930	+	+						++							++		4
S18	S18	AtMYB101	AT2G32460.1	++	+	+	++				++							++		6
S18	S18	AtMYB104	AT2G26950	++	+															2
S19	S19	AtMYB021	AT3G27810	++	+ [bright yellow]	++		++	++			+								6
S19	S19	AtMYB024	AT5G40350	++	+	++	++	++	+			++								7
S20	S20b	AtMYB002	AT2G47190	++	+	++	[light blue]	++	+			++								6
S20	S20a	AtMYB062	AT1G68320	++	++	++	++	++	+		++			++						8

S20	S20b	AtMYB078	AT5G49620.2	++	++	++	++	++	+	++	++			++						9
S20	S20b	AtMYB108	AT3G06490	++	++	++		++	+		++			++						7
S20	S20b	AtMYB112	AT1G48000	++	++	++	++	++	++	++	++			++	++		++			11
S20	S20a	AtMYB116	AT1G25340.1	++	++	++	++	++	+			+			++					8
S21	S21	AtMYB052	AT1G17950	++	++	++	++	++	++	++				++	++	++				10
S21	S21	AtMYB054	AT1G73410	++	++	++	++	++	++	++					++					8
S21	S21	AtMYB056	AT5G17800	++	++	++	++	++	++	++	++				++			++		10
S21	S21	AtMYB069	AT4G33450	++	++	++	++	++		++		+		++				++		9
S21	S21	AtMYB105	AT1G69560	++	+	++		++	++									++		6
S21	S21	AtMYB110	AT3G29020.2	++	+	++	++	++	+		+					++		++		9
S21	S21	AtMYB117	AT1G26780.1	++	+	++		++	+							++				6
**SU**	S21	AtMYB089	AT5G39700	++	+	++	++	++	++	++	++		++	++		++				11
S22	S22	AtMYB044	AT5G67300	++	**++**	**++**	**++**	**++**	+		++			++	**++**			**++**	**++**	11
S22	S22	AtMYB070	AT2G23290	++	++	++	++	++	+					++				++	++	9
S22	S22	AtMYB073	AT4G37260	++	++	++	++	++	+		**++**				++			++	++	10
S22	S22	AtMYB077	AT3G50060	++	**++**	**++**	**++**	++	+					++	**++**		**++**	**++**	**++**	11
S23	S23	AtMYB001	AT3G09230	++	++	++	++	++	+					++						7
S23	S23	AtMYB025	AT2G39880	++	++	++	++	++	+											6
S23	S23	AtMYB109	AT3G55730	++	++	++	++	++	+									++		7
S24	S24	AtMYB053	AT5G65230	++	++	++	++	++	+		++	++	++					++		10
S24	S24	AtMYB093	AT1G34670									+	++							2
S25	S25	AtMYB022	AT5G40430	++	++	++	++	++	++	++								++		8
S25	S25	AtMYB064	AT5G11050	++																1
S25	S25	AtMYB100	AT2G25230	++	++	++	++	++										++		6
S25	S25	AtMYB115	AT5G40360	++	+		++											++		4
S25	S25	AtMYB118	AT3G27785	++	++	++	++	++	++					++				++		8
**SU**	SAt35	AtMYB035	AT3G28470	++	+	++	++	++		++					++				+	8
**SU**	SAt35	AtMYB080	AT5G56110	++		++	++	+		++	++	++	++		++			++		10
**SU**	SAt46	AtMYB046	AT5G12870	++	++		++					**++**	**++**		++			++	+	8
**SU**	SAt46	AtMYB083	AT3G08500	++	+	++	++	++	++	++	**++**		**+**	++		+				11
**SU**	SAt47	AtMYB047	AT1G18710	++	++	++	++	++	++	++	++	+	+	++	++	++	++			14
**SU**	SAt47	AtMYB095	AT1G74430	++	+	++	++	++	++	++	++		++	++	++	++		++		13
**SU**	SAt59	AtMYB027	AT3G53200	++	+	++		+		++	++	+	+	++	++	++		++		12
**SU**	SAt59	AtMYB048	AT3G46130.1	++	++	++	++	++	++	++	++	+	+	++	++	++	+	++		15
**SU**	SAt59	AtMYB059	AT5G59780.3	++	++	++	++	++	++	++	++	+			++	++				11
**SU**	SAt71	AtMYB071	AT3G24310	++	+	++	++	++	+	++	++	++	+		++	++	+	++		14
**SU**	SAt71	AtMYB079	AT4G13480	++	+	++	++	++	++	++	++	+		++	++	++	++			13
**SU**	SAt71	AtMYB121	AT3G30210	++	+	++	++	+	++	++	++	++	++		++	++		++		13
**SU**	SAt85	AtMYB020	AT1G66230	++	+	++	++	+	++	++	++	+	+	++		++				12
**SU**	SAt85	AtMYB040	AT5G14340	++	++	++	++	++	++	++	++	+	+	++	++	++	++			14
**SU**	SAt85	AtMYB042	AT4G12350	++	++	++	++	++	++	++	++	++	++	++	++	++	++			14
**SU**	SAt85	AtMYB043	AT5G16600	++	+	++	++	++	++	++	++	+	+	++	++	++	++			14
**SU**	SAt85	AtMYB085	AT4G22680	++	++	++	++	++		++	++	+	+		++	+	+			12
**SU**	SAt85	AtMYB099	AT5G62320	++	++	++	++	++	++	++	++	++	++		++	++	+			13
**SU**	SAt88	AtMYB088	AT2G02820.2	++	++	++	++	++	++	++	++		+		++	++				10
**SU**	SAt88	AtMYB124	AT1G14350	++	+	++	++	++	++	++	++				++	+	+	++		12
**SU**	SAt91	AtMYB091	AT2G37630	++	++	++	++	++	++	++	++	+	+	++	++	++	++		**+**	15
**SU**	SAt103	AtMYB026	AT3G13890.1	++	+	++	++	++	++	++		+					++	++	+	11
**SU**	SAt103	AtMYB067	AT3G12720	++	+	++	++	++	++	++	++		+	++	++	++	++	++		14
**SU**	SAt103	AtMYB103	AT1G63910	++	+	++	++	++	++	++	**++**	++	**++**		++	++	++	++		14
		**Number of interacting R2R3-MYBs**		**114**	**113**	**112**	**108**	**101**	**86**	**71**	**66**	**62**	**47**	**42**	**51**	**46**	**27**	**52**	**26**	
