# Supplementary material for: Analysis of the DNA-Binding Activities of the Arabidopsis R2R3-MYB Transcription Factor Family by One-Hybrid Experiments in Yeast
Source: PLoS One. 2015 Oct 20;10(10):e0141044. doi: 10.1371/journal.pone.0141044 (PMC4613820; doi:10.1371/journal.pone.0141044)
Supplement: S5 Table — (PDF) [file pone.0141044.s011.pdf]

Table S4. Primers used in this study.

Primers used for their cloning of the representative gene models (P2 to P9 were used for exons amplification from genomic DNA as template).

		Primers		
gene name	gene code	sense	antisense	P2
AtMYB030	AT3G28910	GGAGATAGAACCATGGTGAGGCCTCCTTGTTG	TCCACCTCCGGATCMGAAGAAATTAGTGTTTTCATCCAATAG	
AtMYB031	AT1G74650	GGAGATAGAACCATGGGTAGACCACCTTGTTGC	TCCACCTCCGGATCMTTTTAACTCCTCTAAAGACACGTC	
AtMYB060	AT1G08810.1	GGAGATAGAACCATGGGTAGGCCTCCATGCTG	TCCACCTCCGGATCMAAGCATATTAGAGAGCTCCATCAA	
AtMYB094	AT3G47600	GGAGATAGAACCATGGGAAGACCACCATGCTG	TCCACCTCCGGATCMGAACAACACTTCCTGACCCTC	
AtMYB096	AT5G62470.2	GGAGATAGAACCATGGGAAGACCACCTTGCTG	TCCACCTCCGGATCMGAACATCCCTTCTTGTCCTGC	
AtMYB013	AT1G06180	GGAGATAGAACCATGGGGAGAAGACCATGCTG	TCCACCTCCGGATCMAAACTCAGAAATGTCGGACATTTC	
AtMYB014	AT2G31180	GGAGATAGAACCATGGGAAGAGCACCATGTTGTG	TCCACCTCCGGATCMAAACTCGGGTATGTCGGAAAATTC	
AtMYB015	AT3G23250.1	GGAGATAGAACCATGGGAAGAGCTCCATGCTG	TCCACCTCCGGATCMGAGCCCGGCTAAGAGATCTT	
AtMYB058	AT1G16490	GGAGATAGAACCATGGGCAAAGGAAGAGCACC	TCCACCTCCGGATCMATGTATGAGGAGCTCGTAACTC	
AtMYB063	AT1G79180	GGAGATAGAACCATGGGGAAGGGAAGAGCACC	TCCACCTCCGGATCMATGTATCATGAGCTCGTAGTTCTT	
AtMYB003	AT1G22640	GGAGATAGAACCATGGGAAGATCACCATGCTGC	TCCACCTCCGGATCMATGAGTTCTAACATCAGAAACCCG	CTCTTACCACATCTTTGTAATCCAGCGGCGCGAGGGAGAGATC
AtMYB004	AT4G38620	GGAGATAGAACCATGGGAAGGTCACCGTGCTG	TCCACCTCCGGATCMTTTCATCTCCAAGCTTCGAAAGC	
AtMYB007	AT2G16720	GGAGATAGAACCATGGGAAGATCTCCTTGCTGC	TCCACCTCCGGATCMTTTCATTTCCAAGCTTCGAAAATCC	
AtMYB032	AT4G34990	GGAGATAGAACCATGGGAAGGTCTCCTTGCTG	TCCACCTCCGGATCMTTTCATTTCCAAAGTGCTAAAATCCAA	
AtMYB123	AT5G35550	GGAGATAGAACCATGGGAAAGAGAGCAACTACTAG	TCCACCTCCGGATCMACAAGTGAAGTCTCGGAGCC	
AtMYB075	AT1G56650	GGAGATAGAACCATGGAGGGTTCGTCCAAAGG	TCCACCTCCGGATCMATCAAATTTCACAGTCTCTCCATC	
AtMYB090	AT1G66390	GGAGATAGAACCATGGAGGGTTCGTCCAAAGG	TCCACCTCCGGATCMATCAAGTTCAACAGTCTCTCCATC	CTCTTTCTGCATCGATTTAGCCCAGCTCTCAAAGGAACTTGATG
AtMYB113	AT1G66370	GGAGATAGAACCATGGGCGAATCACCCAAAGG	TCCACCTCCGGATCMATTCAGTTCTAAAGTCTCTTCATCAAA	
AtMYB114	AT1G66380	GGAGATAGAACCATGGAGGGTTCGTCCAAAGG	TCCACCTCCGGATCMAAAAATATCGACTTTTTGGGCCGG	
AtMYB011	AT3G62610	GGAGATAGAACCATGGGAAGGGCGCCGTGC	TCCACCTCCGGATCMAGACAAAAGCCAAGCAGCCATC	CTCTTTCCACATCTCTTTAGCCCGGCATTTTTGGGAAGAGAAC
AtMYB012	AT2G47460	GGAGATAGAACCATGGGAAGAGCGCCATGTTG	TCCACCTCCGGATCMTGACAGAAGCCAAGCGACCA	CTCTTTCCACACCTTTTTAATCCGGCATTTTTGGGGAGAGATC
AtMYB111	AT5G49330	GGAGATAGAACCATGGGAAGGGCTCCGTGTTG	TCCACCTCCGGATCMCTCCAATGTTATCTCTCCAATATC	
AtMYB016	AT5G15310.1	GGAGATAGAACCATGGGTAGATCACCGTGTTGTG	TCCACCTCCGGATCMGAACATCGGTGAATCCGACG	
AtMYB017	AT3G61250	GGAGATAGAACCATGGGAAGAACACCTTGTTGTG	TCCACCTCCGGATCMGAATTTGGAAACCATGGAAACAAG	
AtMYB106	AT3G01140	GGAGATAGAACCATGCCAATCCATGTCAGAGACC	TCCACCTCCGGATCMGAACATCGTCGCGGAATCGG	GCCTTATCACAACATGGCGATATGATCATGATCAGTATGATC
AtMYB009	AT5G16770	GGAGATAGAACCATGGGGCGATCACCATGTTG	TCCACCTCCGGATCMTTCAGGAAATGGCCATGGTTCC	ATTACCGGCTATCGACGACCACTTGTTTCCAAGAAGGGAATGG
AtMYB039	AT4G17785	GGAGATAGAACCATGGGAAGATCACCGTGTTGC	TCCACCTCCGGATCMCCATGGGACAGGTGACGATG	TGACCCGCAATTTTCGACCATTTGTTGCCAAGGAGGGCATG
AtMYB107	AT3G02940	GGAGATAGAACCATGGGGAGATCACCGTGTTG	TCCACCTCCGGATCMTTCACGAAATGGCCAAGGCTC	
AtMYB041	AT4G28110	GGAGATAGAACCATGGGAAGATCACCTTGTTGTG	TCCACCTCCGGATCMAAACATAAAGTCATCTAAGATGAAATCTT	
AtMYB074	AT4G05100	GGAGATAGAACCATGGGACGATCACCATGTTGTG	TCCACCTCCGGATCMTTGGAATTGGAGAAAACCATTAACATC	
AtMYB102	AT4G21440	GGAGATAGAACCATGGCAAGGTCACCTTGTTGC	TCCACCTCCGGATCMTATAATAAAACCATTAACGTCCAAGAAAT	
AtMYB028	AT5G61420.2	GGAGATAGAACCATGTCAAGAAAGCCATGTTGCG	TCCACCTCCGGATCMTATGAAATGCTTTTCAAGCGAGTC	
AtMYB029	AT5G07690	GGAGATAGAACCATGTCAAGAAAGCCATGTTGTGTG	TCCACCTCCGGATCMTATGAAGTTCTTGTCGTCGTAATC	
AtMYB034	AT5G60890	GGAGATAGAACCATGGTGAGGACACCATGTTGC	TCCACCTCCGGATCMGACAAAGACTCCAACCATATTGTC	
AtMYB051	AT1G18570	GGAGATAGAACCATGGTGCGGACACCGTGTTG	TCCACCTCCGGATCMTCCAAAATAGTTATCAATTTCGTCAAAC	
AtMYB076	AT5G07700	GGAGATAGAACCATGTCAAAGAGACCATATTGTATCG	TCCACCTCCGGATCMTAAGAAGTTCTTCTCGTCGGAATC	
AtMYB122	AT1G74080	GGAGATAGAACCATGGTACGGACGCCGTGTTG	TCCACCTCCGGATCMTCCAAAATAATTGTCAATCCCTTCAC	
AtMYB050	AT1G57560	GGAGATAGAACCATGAAGAGACATTCTTGTTGTTACAAA	TCCACCTCCGGATCMAAGGGTCTGACCACCAAAAGAG	
AtMYB055	AT4G01680.1	GGAGATAGAACCATGGGAAGACATTCATGTTGTTAC	TCCACCTCCGGATCMTATATGGCCATATGCATGTTGCTG	
AtMYB061	AT1G09540	GGAGATAGAACCATGGGGAGACATTCTTGCTGTT	TCCACCTCCGGATCMAAGGGACTGACCAAAAGAGACG	
AtMYB086	AT5G26660	GGAGATAGAACCATGGGAAGACATTCTTGTTGTTTTAAG	TCCACCTCCGGATCMAAGGCTCTGATCAAACACCATG	CTCTTTCCACATCTTTGCAAACCTGCGAGTTTAGGGACAGAAC
AtMYB036	AT5G57620	GGAGATAGAACCATGGGAAGAGCTCCATGCTG	TCCACCTCCGGATCMAACACTGTGGTAGCTCATCTGA	CCACATCTCTTCAGCCGCCCAACAATTTTCTGAGGCAGTG
AtMYB037	AT5G23000	GGAGATAGAACCATGGGAAGAGCTCCGTGTTG	TCCACCTCCGGATCMGGAGTAGAAATAGGGCAAGCAT	
AtMYB038	AT2G36890	GGAGATAGAACCATGGGTAGGGCTCCATGTTG	TCCACCTCCGGATCMGTAGTACAACATGAACTTATCCTC	
AtMYB068	AT5G65790	GGAGATAGAACCATGGGAAGAGCACCGTGTTG	TCCACCTCCGGATCMCACATGATTTGGCGCATTGAAG	
AtMYB084	AT3G49690	GGAGATAGAACCATGGGAAGAGCACCGTGTTG	TCCACCTCCGGATCMAAAAAATTGCTTTGAATCAGAATAAACAAG	
AtMYB087	AT4G37780	GGAGATAGAACCATGGGAAGAGCACCGTGCTG	TCCACCTCCGGATCMCTCATTATCGTATAGAGGATAATCAAT	
AtMYB000	AT3G27920	GGAGATAGAACCATGAGAATAAGGAGAAGAGATGAAAAA	TCCACCTCCGGATCMAAGGCAGTACTCAACATCACCA	
AtMYB023	AT5G40330	GGAGATAGAACCATGAGAATGACAAGAGATGGAAAAG	TCCACCTCCGGATCMAAGGCAATACCCATTAGTAAAATCC	
AtMYB066	AT5G14750	GGAGATAGAACCATGAGAAAGAAAGTAAGTAGTAGTGG	TCCACCTCCGGATCMAAAACAGTGTCCATCTATAAAGTCC	
AtMYB018	AT4G25560	GGAGATAGAACCATGGCGAAGACGAAATATGGAG	TCCACCTCCGGATCMCGTCGTTGTTGATGGAGAAAAG	
AtMYB019	AT5G52260	GGAGATAGAACCATGACCAAATCTGGAGAGAGAC	TCCACCTCCGGATCMGACATAATTTTGGTCACTGTTGATG	
AtMYB045	AT3G48920	GGAGATAGAACCATGGTGTTTAAATCAGAAAAATCAAACC	TCCACCTCCGGATCMCATTGGTCGTAGCTCGTTGATG	CTCTTCCCATTCCTCTGCAATCCAGCTTGAAGAGGAATAGTAC
AtMYB033	AT5G06100.2	GGAGATAGAACCATGAGTTACACGAGCACTGACA	TCCACCTCCGGATCMGGGTAGTTCTGTCATTTGACAG	
AtMYB065	AT3G11440	GGAGATAGAACCATGAGTTACACGACGGCGAC	TCCACCTCCGGATCMCAGCGACCAAACAGGAGGC	
AtMYB081	AT2G26960	GGAGATAGAACCATGGGGAAAGTTCGTCAAGATTC	TCCACCTCCGGATCMAAGGATGTGTGTCCCTGATTTG	
AtMYB097	AT4G26930	GGAGATAGAACCATGATCGTGTACGGTGGGGG	TCCACCTCCGGATCMGCAGATCCCTGGCAAGTTGT	GTTATCTGTTCTGCCTGGTAACTGAGCAGCCATGCGAGCCCA
AtMYB101	AT2G32460.1	GGAGATAGAACCATGGATGGTGGTGGAGAGAC	TCCACCTCCGGATCMACAGATGCTAGGCATGTTGCTC	CTCATTGTCTGTTCTTCCAGGTAACTGAGAAGCCATACGAGCCCA
AtMYB104	AT2G26950	GGAGATAGAACCATGATTCAGGATCAAGCGAATGAT	TCCACCTCCGGATCMTATGAATCCCTCATCGAGAAGTAG	GTTATCTGTTCGACCAGGGAATTCTCTAGCCATTTGTGACCA
AtMYB120	AT5G55020	GGAGATAGAACCATGATCATGTACGGAGGAGGAG	TCCACCTCCGGATCMAATCTGCGTTTTGATCATAAACATTTTAC	TCTCGTTGTCTGTTCTTCCCGGTAACTGAGCAGCCATGCGAGCCCATTTG
AtMYB021	AT3G27810	GGAGATAGAACCATGGAGAAAAGAGGAGGAGGAA	TCCACCTCCGGATCMATTACCATTCAATAAATGCATTGATGAC	TCTCGTTGTCTGTTCTTCCCGGTAACTGAGCAGCCATGCGAGCCCATTTG
AtMYB024	AT5G40350	GGAGATAGAACCATGGAGAAAAGAGAAAGTAGTGGT	TCCACCTCCGGATCMATTACCATTATATATATTCATGGGCCA	
AtMYB002	AT2G47190	GGAGATAGAACCATGGAAGATTACGAGCGAATAAAC	TCCACCTCCGGATCMATTATACGAATACGATGTCGTATCG	
AtMYB062	AT1G68320	GGAGATAGAACCATGGAAAATTCGATGAAGAAGAAGAAG	TCCACCTCCGGATCMCTCCCTAAACTGCCAAATGTCA	
AtMYB078	AT5G49620.2	GGAGATAGAACCATGGGTGACAAAGGAAGGAGC	TCCACCTCCGGATCMGAAGCTTCCATTGTCGTGGAC	CTTTTTCCGGTCCTTTTGAGTTCGGCGCAACGAGCGAGAGAG
AtMYB108	AT3G06490	GGAGATAGAACCATGGATGAAAAAGGAAGAAGCTTG	TCCACCTCCGGATCMGAAGCTACCATTATTGTTGAACTG	
AtMYB112	AT1G48000	GGAGATAGAACCATGAATATAAGTAGAACAGAATTCGCA	TCCACCTCCGGATCMCTGTATGAGCCACTTGTTGAGC	
AtMYB116	AT1G25340.1	GGAGATAGAACCATGTCAAATATAACAAAGAAGAAGTGTAA	TCCACCTCCGGATCMATTAAAGTGTGCATACTCGTTAAACTG	
AtMYB052	AT1G17950	GGAGATAGAACCATGATGTGTAGTCGAGGCCATT	TCCACCTCCGGATCMACATAAACCCTGAGAGGCAGAG	
AtMYB054	AT1G73410	GGAGATAGAACCATGATCATGTGCAGCCGAGG	TCCACCTCCGGATCMGGAGGCAGAGTTTCCAACGG	
AtMYB056	AT5G17800	GGAGATAGAACCATGAATCCAAATCTCCTTGAGAAAG	TCCACCTCCGGATCMGGAAGCTCCAACTCCAAGAAAA	
AtMYB069	AT4G33450	GGAGATAGAACCATGGAGATGTCTAGAGGAAGCA	TCCACCTCCGGATCMAGAAGCTAATCCAACACCAAGAAA	
AtMYB105	AT1G69560	GGAGATAGAACCATGGAGATGGTGCATGCTGAC	TCCACCTCCGGATCMCACCGTCCCCAACCCAAG	CATCGAAGCCTACAGCTTTTCCCGGATCTTCCTTGGAGCTTCTC
AtMYB110	AT3G29020.2	GGAGATAGAACCATGAAGATGGATTTTTCATGTTTCCAA	TCCACCTCCGGATCMGTGACCAACTCCTAGAAAATCTAT	CACCTCAATCTGCAGCTCTTTCCTGTTCTTCCTTGCATACTCTC
AtMYB117	AT1G26780.1	GGAGATAGAACCATGTTTATAACGGAAAAACAAGTGTGG	TCCACCTCCGGATCMCGCCGTCCCCAAGCCAAG	
AtMYB044	AT5G67300	GGAGATAGAACCATGGCTGATAGGATCAAAGGTC	TCCACCTCCGGATCMCTCGATTCTCCCAACTCCAATT	
AtMYB070	AT2G23290	GGAGATAGAACCATGTCTGGTTCGACCCGGAA	TCCACCTCCGGATCMCTCGATCCTACCTAATCCAATAAA	
AtMYB073	AT4G37260	GGAGATAGAACCATGTCAAACCCGACCCGTAAG	TCCACCTCCGGATCMCTCCATCTTCCCAATTCCGATT	
AtMYB077	AT3G50060	GGAGATAGAACCATGGCGGATCGTGTTAAAGGTC	TCCACCTCCGGATCMCTCAACCTTAGGTGTTATTACTCC	
AtMYB001	AT3G09230	GGAGATAGAACCATGGAGGCAGAGATCGTGAG	TCCACCTCCGGATCMTGTGGACAGGACATTGGTTATG	
AtMYB025	AT2G39880	GGAGATAGAACCATGAACGGAGAAATCTCTCGTC	TCCACCTCCGGATCMGCTCAAAGCCCTTAAATCTTGTC	
AtMYB109	AT3G55730	GGAGATAGAACCATGGAAGGAGAAACTCATCAATCG	TCCACCTCCGGATCMGCTTCTTGCACAGAAGAAATCTTG	
AtMYB053	AT5G65230	GGAGATAGAACCATGGGAAGATCTCCTAGCTCAG	TCCACCTCCGGATCMAGATTGATAAGAAATGTCTGGAAACAT	
AtMYB092	AT5G10280	GGAGATAGAACCATGGGAAGATCTCCTATCTCTG	TCCACCTCCGGATCMAGGAATGTCGGAAAATATAGAATCATC	

AtMYB093	AT1G34670	GGAGATAGAACCATGGGGAGGTCGCCTTGTTG	TCCACCTCCGGATCMAGATATAACGTTCATGAGGCTTTC	
AtMYB022	AT5G40430	GGAGATAGAACCATGGGTGAACCTCAATTATTTGATG	TCCACCTCCGGATCMATCCCACCAACGCAGCAGC	GCCACCATCTCTTTCAATTTTATGTCCTCAGATTCTGTCCATC
AtMYB064	AT5G11050	GGAGATAGAACCATGGAAGAACAGAAAATTCAAGAGAAG	TCCACCTCCGGATCMGAACAATGGGAACCAAATGTTGC	
AtMYB100	AT2G25230	GGAGATAGAACCATGAAAAAAAATTATCAAAAGAAGAATATAAAG	TCCACCTCCGGATCMTTCCCACCAACGCAGCAGC	
AtMYB115	AT5G40360	GGAGATAGAACCATGTATCACCAAAATCTGATTTCATCG	TCCACCTCCGGATCMATTCCAACCATTCATGAGCATCC	
AtMYB118	AT3G27785	GGAGATAGAACCATGGAGTTCGAGTCAGTGTTCA	TCCACCTCCGGATCMAAGACGACCATGAGCAATCATC	TGGTATTTCATATGTGTGAGGCATCGATGGTTTGTGATGCAC
AtMYB119	AT5G58850	GGAGATAGAACCATGGAAGACCGACGCCTTGT	TCCACCTCCGGATCMGACCAACGGGAAGCAGATGT	CTCACCAACCTTATCAACTTCCTGTCTTCTTCCGCCGTCCAT
AtMYB005	AT3G13540	GGAGATAGAACCATGATGTCATGTGGTGGGAAGA	TCCACCTCCGGATCMGTCATGTCCTAAGCTAGAAGAC	
AtMYB026	AT3G13890.1	GGAGATAGAACCATGGGTCATCACTCATGCTGC	TCCACCTCCGGATCMAGTTATGACGTACTGTCCACAAG	
AtMYB035	AT3G28470	GGAGATAGAACCATGGGAAGACCTCCTTGTTGTG	TCCACCTCCGGATCMATAATCGAAATCATTCAAGAGTTGAGG	
AtMYB046	AT5G12870	GGAGATAGAACCATGAGGAAGCCAGAGGTAGC	TCCACCTCCGGATCMTATGCTTTGTTTGAAGTTGAAGTAAAAC	
AtMYB057	AT3G01530	GGAGATAGAACCATGGAGACGACGATGAAGAAGA	TCCACCTCCGGATCMAATCACATGGTGGTCACCATTAAG	
AtMYB059	AT5G59780.3	GGAGATAGAACCATGAAACTTGTGCAAGAAGAATACC	TCCACCTCCGGATCMAAGGCGACCACTACCATGTTC	
AtMYB072	AT1G56160	GGAGATAGAACCATGGGGAAAGGAAGAGCACC	TCCACCTCCGGATCMTAGACATACTTCTCCGACGAAATT	
AtMYB080	AT5G56110	GGAGATAGAACCATGGGTCGGATTCCATGTTGTG	TCCACCTCCGGATCMAACCATATGATTGATGAGATCATCAG	CTCTTCCCACATCTTTGCAACCCAGCATTCTTAGGGATGAGAC
AtMYB085	AT4G22680	GGAGATAGAACCATGGGGAGACAGCCATGCTG	TCCACCTCCGGATCMAAACCCAAAATCATGAACACCAAAG	
AtMYB088	AT2G02820.2	GGAGATAGAACCATGGAAGAGACAACTAAGCAGAAC	TCCACCTCCGGATCMCAAGCTATCGAGAAGGACTCTT	
AtMYB091	AT2G37630	GGAGATAGAACCATGAAAGAGAGACAACGTTGGAG	TCCACCTCCGGATCMGGGGCGGTCTAATCTGCAAC	
AtMYB098	AT4G18770	GGAGATAGAACCATGGAGAATTTCGTCGACGAGA	TCCACCTCCGGATCMGAGTCCATGAACAAAAGCATCAAT	CTCCACGAGTTGAATCAGTACCCTGTCTTCTTCAGCAGTCCAT
AtMYB099	AT5G62320	GGAGATAGAACCATGGGTGGTCGTAAACCATGTT	TCCACCTCCGGATCMAACATCGAAACATCCAAGTTCTAAATC	CCACTGCAATCTTCGACCATCTATTGCCAAGGCGAGCATGAAG
AtMYB103	AT1G63910	GGAGATAGAACCATGGGTCATCACTCATGCTGC	TCCACCTCCGGATCMAAACGAAGAAGGGAAAGAAGAAGA	
AtMYB124	AT1G14350	GGAGATAGAACCATGGAAGATACGAAGAAGAAAAAGAAG	TCCACCTCCGGATCMCAAGCTATGGAGAAGGACTCTT	
AtMYB125	AT3G60460	GGAGATAGAACCATGGAAGCGAAGAAGGAAGAGA	TCCACCTCCGGATCMAGGACTTGGGATTGGATCAACC	CAGCCGAGAACTTGCATCCATTTTTGAGATTGGGACGGAGT
AtMYB006	AT4G09460	GGAGATAGAACCATGGGAAGATCTCCTTGTTGTG	TCCACCTCCGGATCMCAACAGTGACAAACGCGCCT	
AtMYB008	AT1G35515	GGAGATAGAACCATGGGAAGATCACCATGTTGTG	TCCACCTCCGGATCMGTAACAAGATCCGACCCCGA	CTTTTTCCACAACGCAACAACCCAACGGATTTAGGAAGAGAAC
AtMYB010	AT3G12820	GGAGATAGAACCATGGGGAATAGAAGAGCACCAT	TCCACCTCCGGATCMGCAAGTTTGGTTATCCAAGAAAATG	CTCTTTCCGCATCTCATCAATCCAGCGAGTTTGGGAAGAGATC
AtMYB020	AT1G66230	GGAGATAGAACCATGGGGAGACAACCATGCTG	TCCACCTCCGGATCMAAACAACCCAAAAGTGGAAGAACT	
AtMYB027	AT3G53200	GGAGATAGAACCATGGATTTTAAGAAGGAAGAAACACTT	TCCACCTCCGGATCMTGACCATAGAGAACATGAAAACTC	
AtMYB040	AT5G14340	GGAGATAGAACCATGGGGAGAAAACCGTGCTG	TCCACCTCCGGATCMTAAGAGGAAAAGATTATCATCAAGATTG	
AtMYB042	AT4G12350	GGAGATAGAACCATGGGAAGGCAGCCATGTTG	TCCACCTCCGGATCMGTCCTTGTGACCCGTCTTCA	CTTGCAGCGATCTTGGACCATCTGTTGCCGAGAAGAGCATGAAG
AtMYB043	AT5G16600	GGAGATAGAACCATGGGGAGGCAACCATGTTG	TCCACCTCCGGATCMGAGGAGATCAAATGTCCATGAATC	
AtMYB047	AT1G18710	GGAGATAGAACCATGGGGAGGACGACATGGTT	TCCACCTCCGGATCMAAAGAGATGATCAAGTATGTGACTAC	CTCTTTCCACATCTCTGCAAACCAGCTCTTTGGGGAGGGAAC
AtMYB048	AT3G46130.1	GGAGATAGAACCATGATGCAAGAGGAGGGAAACC	TCCACCTCCGGATCMACCTGACGACCATGGTGATC	CTTTCCTGTTCTGTTCAAACCTGATACTTTTGCTATAAAATC
AtMYB049	AT5G54230	GGAGATAGAACCATGGGAAAATCTTCAAGCTCGG	TCCACCTCCGGATCMTGATAGATTCAAAGCATTATTATTATGATC	
AtMYB067	AT3G12720	GGAGATAGAACCATGAGAGAGAAGTGGGAAATGAAA	TCCACCTCCGGATCMAAAGGTCCAGCTGCCAAGATC	
AtMYB071	AT3G24310	GGAGATAGAACCATGAGTTTGTGGGGAGGGATG	TCCACCTCCGGATCMACAGAAGGGAATGACCATGTTC	
AtMYB079	AT4G13480	GGAGATAGAACCATGGTGGAAGAAGTTTGGAGAAAG	TCCACCTCCGGATCMACAAAATGGAATCACCAAGTTATGG	
AtMYB082	AT5G52600	GGAGATAGAACCATGGAATGCAAAAGAGAAGAAGGG	TCCACCTCCGGATCMAAGCAGAGGAAAGAAATCGGTAAA	CTTTTTCCTCCTCTCTTCAACCCGGATCTACGAGAAATGTCTG
AtMYB083	AT3G08500	GGAGATAGAACCATGATGATGAGGAAACCGGACA	TCCACCTCCGGATCMATCGACTTGGAAATCAAGGAAGG	CGAGTAGCTATTTGAGACCACCTGTTACCAAGAATGGAATG
AtMYB089	AT5G39700	GGAGATAGAACCATGTATCTGTTTATGTATAAATGTAACATAG	TCCACCTCCGGATCMTTGTTTAGTATTAAGGAGACACAAACC	CACCTTTGTCTACAACTTAGACTTGTTCTTGGTTCCATCTTCTC
AtMYB095	AT1G74430	GGAGATAGAACCATGGGGAGGACGACGTGG	TCCACCTCCGGATCMAAGAAGGAACAGGTCAAGGTGT	
AtMYB121	AT3G30210	GGAGATAGAACCATGCTTGATTGGGGAGTTCAAG	TCCACCTCCGGATCMAAAAATATAGCCTCCCATGTAAACTC	

Primers used for their cloning of the different DNA motifs analysed in this study

Sequence (F)	Sequence (R)	Forward primers (5' to 3')	Reverse primers (5' to 3')
CACCAACC	*GGTTGGTG*	CGACAAGAACACCACCAACCCACCAACCCACCAACCCACCAACCCACCAACCCACCAACC	GAAGAAGAGCACGGTTGGTGGGTTGGTGGGTTGGTGGGTTGGTGGGTTGGTGGGTTGGTG
CACCTACC	*GGTAGGTG*	CGACAAGAACACCACCTACCCACCTACCCACCTACCCACCTACCCACCTACCCACCTACC	GAAGAAGAGCACGGTAGGTGGGTAGGTGGGTAGGTGGGTAGGTGGGTAGGTGGGTAGGTG
TAACTA	*TAGTTA*	CGACAAGAACACTAACTATAACTATAACTATAACTATAACTATAACTA	GAAGAAGAGCACTAGTTATAGTTATAGTTATAGTTATAGTTATAGTTA
TAACCT	*AGGTTA*	CGACAAGAACACTAACCTTAACCTTAACCTTAACCTTAACCTTAACCT	GAAGAAGAGCACAGGTTAAGGTTAAGGTTAAGGTTAAGGTTAAGGTTA
ACAAGTTA	*TAACTTGA*	CGACAAGAACACTAACTTGTTAACTTGTTAACTTGTTAACTTGTTAACTTGTTAACTTGT	GAAGAAGAGCACACAAGTTAACAAGTTAACAAGTTAACAAGTTAACAAGTTAACAAGTTA
AGTGGTTA	*TAACCACT*	CGACAAGAACACAGTGGTTAAGTGGTTAAGTGGTTAAGTGGTTAAGTGGTTAAGTGGTTA	GAAGAAGAGCACTAACCACTTAACCACTTAACCACTTAACCACTTAACCACTTAACCACT
GACAGTTG	*CAACTGTC*	CGACAAGAACACCAACTGTCCAACTGTCCAACTGTCCAACTGTCCAACTGTCCAACTGTC	GAAGAAGAGCACGACAGTTGGACAGTTGGACAGTTGGACAGTTGGACAGTTGGACAGTTG
TACTGTTG	*CAACAGTA*	CGACAAGAACACTACTGTTGTACTGTTGTACTGTTGTACTGTTGTACTGTTGTACTGTTG	GAAGAAGAGCACCAACAGTACAACAGTACAACAGTACAACAGTACAACAGTACAACAGTA
TGCGGTTG	*CAACCGCA*	CGACAAGAACACTGCGGTTGTGCGGTTGTGCGGTTGTGCGGTTGTGCGGTTGTGCGGTTG	GAAGAAGAGCACCAACCGCACAACCGCACAACCGCACAACCGCACAACCGCACAACCGCA
AGTAGTTA	*TAACTACT*	CGACAAGAACACAGTAGTTAAGTAGTTAAGTAGTTAAGTAGTTAAGTAGTTAAGTAGTTA	GAAGAAGAGCACTAACTACTTAACTACTTAACTACTTAACTACTTAACTACTTAACTACT
ATTAGTTG	*CAACTAAT*	CGACAAGAACACCAACTAATCAACTAATCAACTAATCAACTAATCAACTAATCAACTAAT	GAAGAAGAGCACATTAGTTGATTAGTTGATTAGTTGATTAGTTGATTAGTTGATTAGTTG
AAAAGTTA	*TAACTTTT*	CGACAAGAACACAAAAGTTAAAAAGTTAAAAAGTTAAAAAGTTAAAAAGTTAAAAAGTTA	GAAGAAGAGCACTAACTTTTTAACTTTTTAACTTTTTAACTTTTTAACTTTTTAACTTTT
GTCAGTTA	*TAACTGAC*	CGACAAGAACACGTCAGTTAGTCAGTTAGTCAGTTAGTCAGTTAGTCAGTTAGTCAGTTA	GAAGAAGAGCACTAACTGACTAACTGACTAACTGACTAACTGACTAACTGACTAACTGAC
GTTAGTTG	*CAACTAAC*	CGACAAGAACACGTTAGTTGGTTAGTTGGTTAGTTGGTTAGTTGGTTAGTTGGTTAGTTG	GAAGAAGAGCACCAACTAACCAACTAACCAACTAACCAACTAACCAACTAACCAACTAAC
GCTTGTTG	*CAACAAGC*	CGACAAGAACACCAACAAGCCAACAAGCCAACAAGCCAACAAGCCAACAAGCCAACAAGC	GAAGAAGAGCACGCTTGTTGGCTTGTTGGCTTGTTGGCTTGTTGGCTTGTTGGCTTGTTG
GTTAGTTA	*TAACTAAC*	CGACAAGAACACGTTAGTTAGTTAGTTAGTTAGTTAGTTAGTTAGTTAGTTAGTTAGTTA	GAAGAAGAGCACTAACTAACTAACTAACTAACTAACTAACTAACTAACTAACTAACTAAC

LIC primer for the generation of the pHisi-LIC vector (5' to 3')

pHisi-LIC-For	*AATTCGACAAGAACACGTGCTCTTCTTCAA*
pHisi-LIC-Rev	*CTAGTTGAAGAAGAGCACGTGTTCTTGTCG*

P3	P4	P5	P6	P7
GATCTCTCCCTCGCGCCGCTGGATTACAAAGATGTGGTAAGAG	CTCCCAGCTATTAAAGACCATTTGTTACCGAGCAAGCTATGG	CCATAGCTTGCTCGGTAACAAATGGTCTTTAATAGCTGGGAG		
CATCAAGTTCCTTTGAGAGCTGGGCTAAATCGATGCAGAAAGAG	CGACCAGCAATCAAGGACCACCTATTTCCTAGAAGCTTATGA	TCATAAGCTTCTAGGAAATAGGTGGTCCTTGATTGCTGGTCG		
GTTCTCTTCCCAAAAATGCCGGGCTAAAGAGATGTGGAAAGAG	TTGCTCGCAATTGTTGACCACCTGGTTCCCAAAGTGGAATGC	GCATTCCACTTTGGGAACCAGGTGGTCAACAATTGCGAGCAA		
GATCTCTCCCCAAAAATGCCGGATTAAAAAGGTGTGGAAAGAG	TGACCCGCGATTAGTGACCACCTGTTTCCCAAAGTGGAATGC	GCATTCCACTTTGGGAAACAGGTGGTCACTAATCGCGGGTCA		
GATCATACTGATCATGATCATATCGCCATGTTGTGATAAGGC	CTCTTTCCACACCTTTGGAGACCGGCTTTCTCAGGCAAAGAAC	GTTCTTTGCCTGAGAAAGCCGGTCTCCAAAGGTGTGGAAAGAG	TGAGTTGCAATCGCTGACCACCTGTTTCCGAGGAGAGCGTGG	CCACGCTCTCCTCGGAAACAGGTGGTCAGCGATTGCAACTCA
**CCATTCCCTTCTTGGAAACAAGTGGTCGTCGATAGCCGGTAAT**	CCATGGTTCCGAACAAGTTTGCTCTATTATCTCTTTCCAAAAAG	CTTTTTGGAAAGAGATAATAGAGCAAACTTGTTCGGAACCATGG		
CATGCCCTCCTTGGCAACAAATGGTCGAAAATTGCGGGTCA	GACGATGTGGGTGACGTTAAGTCTAAGAAACTTTTCCAGCAAG	CTTGCTGGAAAAGTTTCTTAGACTTAACGTCACCCACATCGTC		
GTTCTGTCCCTAAACTCGCAGGTTTGCAAAGATGTGGAAAGAG	CGCGTTGCGATTTGAGACCATCTGTTGCCTAATGCAGCATGG	CCATGCTGCATTAGGCAACAGATGGTCTCAAATCGCAACGCG		
CACTGCCTCAGAAAATTGTTGGGCGGCTGAAGAGATGTGG	CTGAGCTGCAATTATGGACCACCACCGGCTTCCAATGCTAATGTAG	CTACATTAGCATTGGAAGCCGGTGGTGGTCCATAATTGCAGCTCAG		
GTACTATTCCTCTTCAAGCTGGATTGCAGAGGAATGGGAAGAG	GAATTTCGATATCTGAGACCATTTGTTACCCAACATGGAATGA	TCATTCCATGTTGGGTAACAAATGGTCTCAGATATCGAAATTC		
TGGGCTCGCATGGCTGCTCAGTTACCAGGCAGAACAGATAAC	CATCTTCCTTTGGTGTTAAACCTTGCAAGGAACATAGACTGTTG	CAACAGTCTATGTTCCTTGCAAGGTTTAACACCAAAGGAAGATG		
TGGGCTCGTATGGCTTCTCAGTTACCTGGAAGAACAGACAATGAG	CTTGTTGTGAATATTAGGGTTTGCTCCCAATGATGAATGATTAGAAG	CTTCTAATCATTCATCATTGGGAGCAAACCCTAATATTCACAACAAG		
TGGTCACAAATGGCTAGAGAATTCCCTGGTCGAACAGATAAC	CTCTCATTTGAACCAACATAACCAGTTGTGTTTGCAATGTCTG	CAGACATTGCAAACACAACTGGTTATGTTGGTTCAAATGAGAG		
CAAATGGGCTCGCATGGCTGCTCAGTTACCGGGAAGAACAGACAACGAGA				
CAAATGGGCTCGCATGGCTGCTCAGTTACCGGGAAGAACAGACAACGAGA				
CTCTCTCGCTCGTTGCGCCGAACTCAAAAGGACCGGAAAAAG	ACTTGTGATCAAATTCATTACTATTGCCCCAACGTGTGTG	CACACACGTTGGGGCAATAGTAATGAATTTGATCACAAGT		
GAGAAGCTCCAAGGAAGATCCGGGAAAAGCTGTAGGCTTCGATG	CTCTCATTTTCATGATCATAACCTGGGAAGCAAGGAACTGGTA	TACCAGTTCCTTGCTTCCCAGGTTATGATCATGAAAATGAGAG		
GAGAGTATGCAAGGAAGAACAGGAAAGAGCTGCAGATTGAGGTG	CTTCATCATCATCTACATTACCAGGAGAAAGATTGAAGATTTTG	CAAAATCTTCAATCTTTCTCCTGGTAATGTAGATGATGATGAAG		

GATGGACAGAATCTGAGGACATAAAATTGAAAGAGATGGTGGC	CTTCTTCACTCCAAGTAGTTTTCTTAACGTTAGGACGAGCATG	CATGCTCGTCCTAACGTTAAGAAAACTACTTGGAGTGAAGAAG		
GTGCATCACAAACCATCGATGCCTCACACATATGAAATACCA	GTCCACTAGCTGCACCAATAACTTGTCTTCTTCAGGAGTCCAT	ATGGACTCCTGAAGAAGACAAGTTATTGGTGCAGCTAGTGGAC	CTTCTTCAGTCCATCCATCTTTCTTGATATCGGGACGGAGATG	CATCTCCGTCCCGATATCAAGAAAGATGGATGGACTGAAGAAG
ATGGACGGCGGAAGAAGACAGGAAGTTGATAAGGTTGGTGAG	CTTCTTCACTCCATCCATCTTTCTTAATATCAGGACGGAGATG	CATCTCCGTCCTGATATTAAGAAAGATGGATGGAGTGAAGAAG		
GTCTCATCCCTAAGAATGCTGGGTTGCAAAGATGTGGGAAGAG	TGCGCCGCAATCAACGACCACCGGTTACCAAGAACAGAGTGA	TCACTCTGTTCTTGGTAACCGGTGGTCGTTGATTGCGGCGCA		
ATGGACTGCTGAAGAAGACAGGGTACTGATTCAACTCGTGGAG	CTTCTTCACTCCATGTTTCTTTCTTAATGTCAGGTCTCAAATG	CATTTGAGACCTGACATTAAGAAAGAAACATGGAGTGAAGAAG		
CTTCATGCTCGCCTTGGCAATAGATGGTCGAAGATTGCAGTGG				
ACTCCGTCCCAATCTCAAAAATGGATGCAAGTTCTCGGCTG	CTTCCTCAACCAAACCAAAACCCTGAGAGGATTGACGGATTGG	CCAATCCGTCAATCCTCTCAGGGTTTTGGTTTGGTTGAGGAAG		
GTTCTCTTCCTAAATCCGTTGGGTTGTTGCGTTGTGGAAAAAG	CATCCATGCTGGAAAAGCCATTTGTTGCCAAATAAACTATGA	TCATAGTTTATTTGGCAACAAATGGCTTTTCCAGCATGGATG	AAGGCAATTGATTCCTGAAATCTTAGCTAGCCTACCGCCGCT	AGCGGCGGTAGGCTAGCTAAGATTTCAGGAATCAATTGCCTT
GATCTCTTCCCAAACTCGCTGGATTGATGAGATGCGGAAAGAG	GTTGGATGCGATCTTAGACCACTTGTTTCCATAAGCTTGGTGA	TCACCAAGCTTATGGAAACAAGTGGTCTAAGATCGCATCCAAC		
CTTCATGCTCTTCTCGGCAACAGATGGTCCAAGATCGCTGCAAG				
GTTCCCTCCCCAAAAGAGCTGGTTTGCAGAGATGTGGAAAGAG	CTTCTTCGCCATGGCTGCCCACCTGTTTCCGAGAACAGCATGA	TCATGCTGTTCTCGGAAACAGGTGGGCAGCCATGGCGAAGAAG		
GATTTTATAGCAAAAGTATCAGGTTTGAACAGAACAGGAAAG	TTCGGGCTATTTTCGACCACCTGTTTCCCCACTTAGCGTG	CACGCTAAGTGGGGAAACAGGTGGTCGAAAATAGCCCGAA		
CAGACATTTCTCGTAGATCCGGGTTGAAGAGAGGAGGAAAAAG	CGACCAGCGATCAACGACCATCTGTTTCCAAGAAGCTTATGC	GCATAAGCTTCTTGGAAACAGATGGTCGTTGATCGCTGGTCG		
CATTCCATTCTTGGTAACAGGTGGTCTCAAATAGCTACTCG				
GAGAAGATGGAACCAAGAACAAGTCTAAGTTGTAGACAAAGGTG				

P8	P9
CAAGCTGTTGGTCTCCGTTTCCTTGGTTTGGTTTAGTCCAGT	ACTGGACTAAACCAAACCAAGGAAACGGAGACCAACAGCTTG

TTCCCAGCAATCAAAGACCATTTAGCAAATTTGGAAAGGAAAG	CTTTCCTTTCCAAATTTGCTAAATGGTCTTTGATTGCTGGGAA
